# Environmental signals perceived by the brain abate pro-metastatic monocytes by dampening glucocorticoids receptor signaling

**DOI:** 10.1186/s12935-023-02855-4

**Published:** 2023-02-01

**Authors:** María Magdalena Canali, Mélanie Guyot, Thomas Simon, Douglas Daoudlarian, Joelle Chabry, Clara Panzolini, Agnès Petit-Paitel, Nicolas Hypolite, Sarah Nicolas, Pierre Bourdely, Heidy Schmid-Antomarchi, Annie Schmid-Alliana, Javier Soria, Babou Karimdjee Soilihi, Paul Hofman, Armelle Prevost-Blondel, Masashi Kato, Evelyne Mougneau, Nicolas Glaichenhaus, Philippe Blancou

**Affiliations:** 1 Molecular and Cellular Pharmacology Institute, Université Côte d’Azur, CNRS, 660 Route des Lucioles, Valbonne, France; 2 Université Côte d’Azur, CNRS, INSERM, Valrose Biology Institute, 28 Avenue de Valombrose, Nice, France; 3 Laboratory of Clinical and Experimental Pathology and Biobank, Nice University Hospital, Nice, France; 4 Research Institute on Cancer and Aging, Université Côte d’Azur, CNRS, INSERM, 28 Avenue de Valombrose, Nice, France; 5 Université Paris Descartes, CNRS, INSERM, Institut Cochin, 22 rue Méchain, 75014 Paris, France; 6 Department of Occupational and Environmental Health, Nagoya University Graduate School of Medicine, Nagoya, Aichi Japan; 7 Polyclinique Saint Jean, Cagnes sur mer, France

## Abstract

While positive social-behavioral factors predict longer survival in cancer patients, the underlying mechanisms are unknown. Since tumor metastasis are the major cancer mortality factor, we investigated how an enriched environment (EE) conductive to enhanced sensory, cognitive and motor stimulation impact metastatic progression in lungs following intravasation in the circulation. We find that mice housed in EE exhibited reduced number of lung metastatic foci compared to control mice housed in a standard environment (SE). Compared to SE mice, EE mice increased lung inflammation as early as 4 days after circulating tumor cells extravasation. The impact of environmental signals on lung metastasis is independent of adrenergic receptors signaling. By contrast, we find that serum corticosterone levels are lower in EE mice and that glucocorticoid receptor (GR) antagonist reduces the number of lung metastasis in SE mice. In addition, the difference of the number of lung metastasis between SE and EE mice is abolished when inflammatory monocytes are rendered deficient in GR signaling. This decreased GR signaling in inflammatory monocytes of SE mice results in an exacerbated inflammatory profile in the lung. Our study shows that not only EE reduces late stages of metastatic progression in lungs but disclose a novel anti-tumor mechanism whereby GR-dependent reprogramming of inflammatory monocytes can inhibit metastatic progression in lungs. Moreover, while inflammatory monocytes have been shown to promote cancer progression, they also have an anti-tumor effect, suggesting that their role is more complex than currently thought.

**Supplementary Information:**

The online version contains supplementary material available at 10.1186/s12935-023-02855-4.

## Introduction

Since most cancer patients die from metastatic tumors, the understanding of the early steps of metastatic progression will be critical for the development of new therapies to improve patient outcome [1]. Experimental studies in rodents have helped to elucidate some of the mechanisms by which malignant cells metastasize from primary solid tumors in the lungs, liver, bone and brain [2, 3]. To disseminate, cancer cells undertake several steps known as the metastatic cascade. These steps are not necessarily sequential, but they are essential conditions cancer cell must meet to metastasize. First, cancer cells escape from the tumoricidal immune response that is mainly mediated by CD8^+^ T-cells and natural killer (NK) cells. Tumor cells also must change the microenvironment of the primary site to increase the density of blood vessels (angiogenesis), which will ultimately lead to tumor cell egress by invasion through the surrounding stroma and intrusion into blood vessels (intravasation). They eventually produce factors like cytokines, growth factors, metabolites, among others; that establish a tumor-supportive environment (pre-metastatic niche) in the future metastatic site. The circulating tumor cells (CTCs) are then arrested in microvessels in the metastatic site where they need also to survive and proliferate to form the deadly metastatic tumor. Each step of the metastatic cascade can be regulated by innate immune cells, including monocytes, neutrophils, NK cells and macrophages [2, 3]. In particular, inflammatory monocytes (IMs), which are recruited and differentiate very early in the metastatic site, are key players in cancer metastasis through promotion of tumor cell extravasation, growth, and angiogenesis [4]. Differentiation of IMs and their subsequent pro- or anti-tumorigenic role not only depends upon tumor- or stromal- cell signaling in tumor microenvironment but also on brain-derived signals.

A large body of evidence suggests that the social and physical environment in which we live has a profound impact on cancer incidence or progression [5, 6]. For example, epidemiological studies have shown that psychological stress, chronic depression and lack of social support are risk factors for cancer progression [7, 8]. By contrast, positive factors such as social support and optimism predict longer survival [9, 10]. In agreement with human studies, chronic stress increases tumor progression in mouse cancer model [11–19]. In contrast, mice housed in an enriched environment (EE), in which they experience enhanced levels of sensory, cognitive and motor stimulation, exhibit delayed growth of tumors [20] and increased survival in melanoma [21], colon [21] and glioma cancer models [22]. However, these studies were restricted to primary tumor growth and did not investigate the impact of EE on tumor metastasis.

In response to environmental stimuli, the central nervous system signals for adaptive changes in physiological functions via the release of neuroeffector molecules (such as noradrenaline) from nerves of the sympathetic nervous system (SNS) or glucocorticoids (GCs) from the hypothalamic-pituitary-adrenal (HPA) axis [23, 24,25]. SNS activation promotes metastasis of solid tumors by stimulating macrophage infiltration, inflammation, angiogenesis, epithelial-mesenchymal transition and tumor invasion, and by inhibiting cellular immune responses and programmed cell death [26]. While HPA activation and increased glucocorticoid signaling also contribute to cancer progression, only the GC receptors (GRs) signaling on non-immune cells has been documented as the main promotor of tumor growth and metastasis [27].

As immune cells express both GC receptors (GRs) and adrenergic receptors (ARs), environmental signals perceived by the brain could theoretically regulate each step of metastatic process, including the extravasation and seeding steps, by interfering with signaling through these receptors on immune cells. The goal of this study was to test whether and how environmental signals perceived by the brain could affect tumor metastasis using the well-characterized mouse lung metastasis model.

## Results

### Environmental signals perceived by the brain protect from metastasis

As a measure of positive-stress-induced changes in the brain we evaluated several behavioral and biological parameters. In agreement with previous studies [20], we found that EE mice exhibited decreased anxiety, increased neurogenesis and exploratory activity, as well as enhanced learning and memory compared to mice housed in a standard environment (SE) (Additional file 1: Fig. S1).

**Fig. 1:**
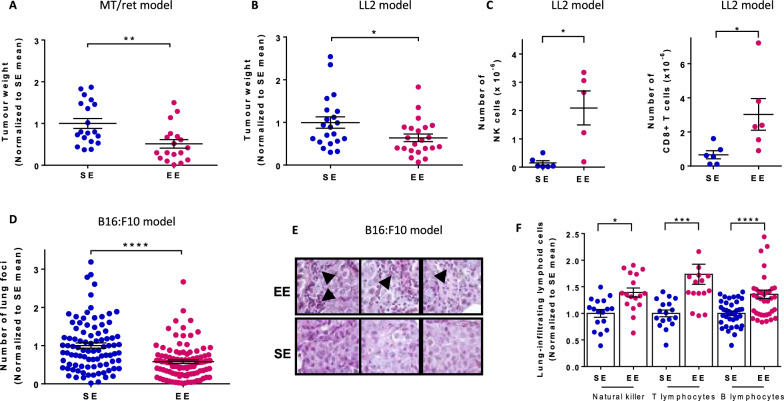
Environmental signals impact the late stages of the metastatic process. MT/ret (**A**) and wt (**B**–**F**) mice were housed for 10–12 weeks under SE or EE conditions. **A** Mice were sacrificed at the age of 3 months and distant tumors were dissected out. Tumor weight in individual mice normalized to the mean tumor weight determined in SE mice (n = 19 for SE, n = 18 for EE). **B** and **C** LL2 cells were injected into the portal vein and liver tumors were dissected out 21 days later. **B** Tumor weight in individual mice normalized to the mean tumor weight determined in SE mice (n = 21 for SE, n = 22 for EE). **C** Number of tumor-infiltrating NK and CD8^+^ cells in individual mice (n = 5 for SE, n = 5 for EE). **D**–**F** Mice were injected with B16:F10 cells into the tail vein and analyzed 14 days later for numbers of lung metastatic foci (**D**) and tumor-infiltrating immune cells (E and F). **D** Number of lung foci in individual mice normalized to the mean value determined in SE mice (n = 91 for SE, n = 98 for EE). **E** Representative HES-staining of lung sections from SE and EE mice. Arrows indicate infiltrating lymphoid cells. **F** Number of lung-infiltrating NK, T and B cells in individual mice normalized to the mean number of lymphoid cells in SE mice (n = 17 for SE and EE). Mean ± s.e.m. of three (**A**, **B**), eight (**D**) and two (**C**, **F**) experiments. *p < 0.05; **p < 0.01; ***p < 0.001; ****p < 0.0001; *ns* not significant

To investigate the impact of positive environmental stimuli on tumor metastasis, we first used MT/ret mice [28] which spontaneously develop a primary uveal tumor before the age of 3 weeks and subsequently cutaneous and distant metastases [29]. MT/ret mice examined at 12 weeks of age showed a marked reduction (40%) in metastatic tumor weight when housed in EE (Fig. 1A). Since primary tumor cell growth can be affected by factors other than anti-tumor immune response in EE housing [21, 22], we used two models of metastatic tumors in which wild-type (wt) mice are injected with cancer cells to avoid direct effects over the primary tumor. In the Lewis Lung Carcinoma (LL2) liver metastatic model, tumor weight was decreased in EE mice (Fig. 1B) and the numbers of tumor-infiltrating CD8 + and NK cells were increased (Fig. 1C). In the melanoma B16:F10 lung metastatic model, the number of lung foci was reduced by nearly 40% in EE mice (Fig. 1D) and, at variance with SE mice, tumor-infiltrating lymphoid cells were consistently detected (Fig. 1E). As in the LL2 liver model, the numbers of lung-infiltrated NK, T and B cells were increased in EE mice compared to SE mice (Fig. 1F). Thus, in three different models, EE protects from cancer metastasis and protection is associated with increased numbers of tumor-infiltrating immune cells. Since B16:F10 lung model involves low stress intervention with minimally invasive procedures, we decided to delineate the mechanism of EE-mediated metastasis protection in this model.

### EE housing decreases metastasis at early time points while increasing immune infiltration

Based on the hypothesis that the immune response will be differentially regulated in SE and EE conditions, we assessed cellular infiltrate and cytokine production at different time points. Before any intervention, neither the frequencies of the main immune cell types (Additional file 1: Fig. S2) nor the levels of cytokines and chemokines (Additional file 1: Fig. S3) were different between SE and EE mice.

**Fig. 2:**
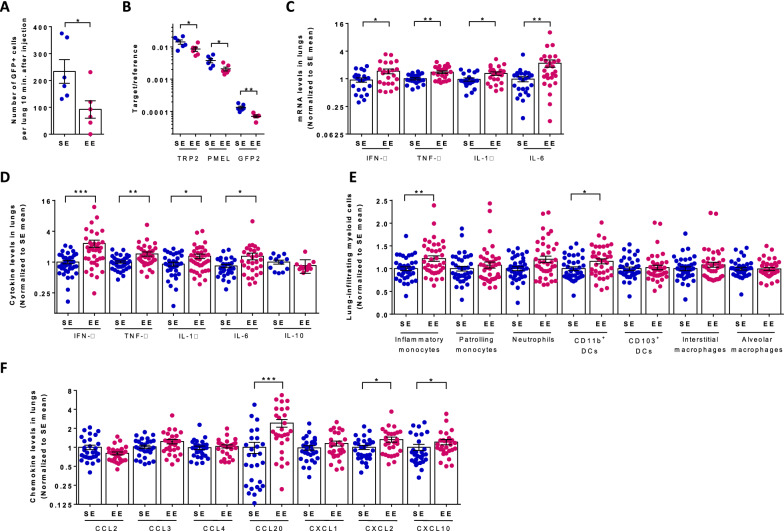
Lung tumor cells, chemokines, cytokines and immune cell types in SE and EE mice. SE and EE mice were injected with either GFP-expressing B16:F10 cells (**A**, **B**, n = 6 for SE, n = 6 for EE) or B16:F10 cells [**C–**F, n = 24 for SE, n = 26 for EE)] and sacrificed 4 days later. Lung cells were analyzed by flow cytometry for GFP^+^ cells (**A**) or the indicated immune cell types (**E**, **F**). Lung RNAs were analyzed for the indicated tumor-specific (**B**) and cytokine (**C**) genes by quantitative RT-PCR. Lung protein extracts were analyzed for the indicated cytokines (**D**) and chemokines (**G**). **A** Number of GFP^+^ cells in individual mice. **B** Ratio between TRP2, PMEL and GFP2 (target) and GAPDH (reference) mRNA levels in individual mice on day 4. **C** IFN-γ, TNF-α, IL-1β, IL-6 mRNA levels in individual mice normalized to the mean levels determined in SE mice. **D** IFN-γ, TNF-α, IL-1β, IL-6 and IL-10 levels in lungs in individual mice normalized to the mean levels determined in SE mice. Mean cytokine levels in SE mice were 17.6 pg/ml (IFN-γ), 338.2 pg/ml (TNF-α), 459.3 pg/mg (IL-1β, 1008.5 pg/ml (IL-6) and 6.42 pg/mg (IL-10). **E** Number of myeloid cells normalized to the mean values determined in SE mice. Inflammatory monocytes (Siglec-F^−^ CD11c^−^ Ly6G^−^ CD11b^high^ MHCII^−^ CD64^−^ Ly6C^+^), patrolling monocytes (Siglec-F^−^ CD11c^−^ Ly6G^−^ CD11b^high^ MHCII^−^ CD64^−^ Ly6C^−^), neutrophils (Siglec-F^−^ CD11c^−^ CD103^−^ CD11b^+^ Ly6G^high^), CD11b^+^ DCs (Siglec-F^−^ CD11c^+^ CD11b^high^ MHCII^+^ CD64^−^ CD24^+^), CD103^+^ DCs (Siglec-F^−^ CD11b^−^ CD103^+^ CD11c^+^ CD24^+^), interstitial macrophages (Siglec-F^−^ CD11c^−^ CD11b^high^ MHCII^+^ CD64^+^ CD24^−^) and alveolar macrophages (CD11b^−^ Siglec-F^+^ CD11c^+^ CD64^+^–) were identified by flow cytometry based on the indicated surface markers. **F** Chemokine levels in individual mice normalized to the mean levels determined in SE mice. Mean chemokine levels in SE mice were 6164 pg/mg (CCL2), 1336 pg/mg (CCL3), 2745 pg/ml (CCL4), 2867 pg/mg (CCL20), 560 pg/mg (CXCL1), 230 pg/ml (CXCL2) and 1647 pg/ml (CXCL10). Mean ± s.e.m. of two (A, B) and three (CG) experiments. *p,  < 0.05; **p,  < 0.01; ***p,  < 0.001; ****p,  < 0.0001

Cameron et al. showed that intravenously infused B16:F10 cells are rapidly trapped in lung vessels and extravasate into the lung parenchyma [30]. While most tumor cells die within the first 4 days or remain dormant, a few divides to develop into macroscopic tumors. They also demonstrated that the number of cells found at day 4 were predictive of the number of metastatic foci at day 15. To investigate the mechanisms underlying the impact of environmental signals on lung metastasis, we injected mice with GFP-expressing B16:F10 cells and analyzed their lungs 4 days later. EE mice had reduced numbers of GFP^+^ tumor cells (Fig. 2A) and lower levels of melanoma-specific and gfp mRNA in lung tissue (Fig. 2B). Therefore, the difference between EE and SE seems to be established before day 4, rising two possible explanations: either lung-infiltrating CTCs are rapidly killed after extravasation, or their division is rapidly inhibited in EE mice.

We next analyzed the lungs of SE and EE mice for both cytokine and chemokine levels and numbers of infiltrating immune cell types at day 4. The lungs of EE mice showed higher levels of IFN-γ, TNF-α, IL-1β, IL-6, mRNA (Fig. 2C) and protein (Fig. 2D). By contrast, IL-10 serum levels were not changed between SE and EE (Fig. 2D). At that time, EE mice exhibited increased numbers of both iMonos and CD11b^+^ DCs as detected by flow cytometry (Fig. 2E). In agreement with the higher numbers of T, B and NK cells in lungs form EE mice relative to SE mice (Fig. 1F), lung protein extracts from EE mice also contained higher levels of CCL20, CXCL2 and CXCL10 that are all chemotactic for these cells (Fig. 2F).

### The impact of environmental signals on lung metastasis is independent on signaling through adrenergic receptors

**Fig. 3:**
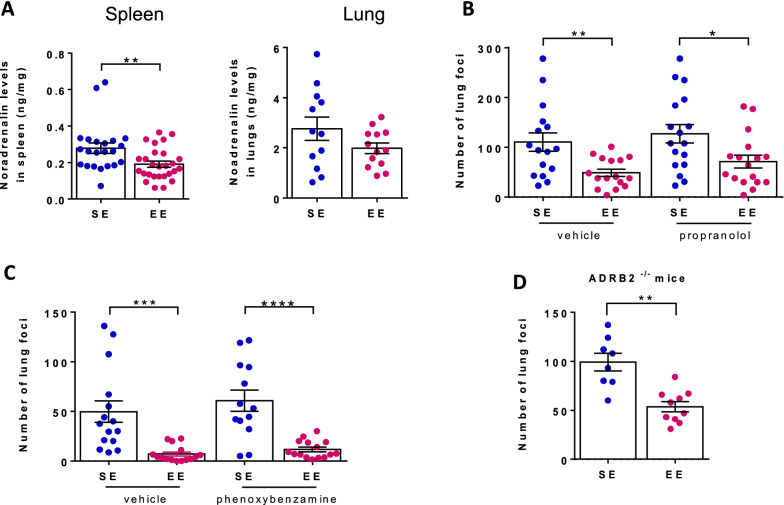
The impact of environmental signals on lung metastasis is not mediated by adrenergic receptors. Wt (**A**, **B**, **C**) or β2-AR-deficient ADRB2^−/−^ (**D**) mice were housed under SE or EE conditions for 10–12 weeks. **A** Spleen (left, n = 22 for SE, n = 30 for EE) and lung (right, n = 12 for SE, n = 13 for EE) noradrenaline levels in individual mice. **B**, **C**, **D** Mice were injected with B16:F10 cells and either treated with propranolol (**B**, n = 16–17 for SE and EE), phenoxybenzamine (**C**, n = 13–15 for SE, n = 15–18 for EE) or vehicle (**B**, **C**) or left untreated (**D**, n = 8 for SE, n = 10 for EE). **B–D** Number of lung foci normalized to SE mean in individual mice two weeks after tumor cell injection. Mean ± s.e.m. of two experiments (**A**–**D**). *p < 0.05; **p < 0.01; ***p < 0.001; ****p < 0.0001

SNS signaling through α- and β-adrenergic receptors regulates a wide variety of molecular processes involved in tumor metastasis, including expression of pro-inflammatory mediators by tumor cells and immune cells, recruitment and pro-metastatic transcriptional re-programming of macrophages, angiogenesis, and inhibition of cytokines and cytotoxic function in adaptive immune responses [25]. We found that noradrenaline levels were reduced in EE mice relative to SE mice in spleen (0.28 ± 0.03 in SE mice versus 0.19 ± 0.02 in EE mice, p < 0.01) but not in lungs (2.76 ± 0.46 in SE mice versus 1.99 ± 0.21 in EE mice, p > 0.05) (Fig. 3A). Most importantly, the difference in the number of lung foci in SE and EE mice was neither abolished by the pharmacological inhibition of β1/2-ARs using propranolol (Fig. 3B), nor by the pharmacological inhibition of α-ARs using phenoxybenzamine (Fig. 3C). In agreement with the results of β1/2-AR pharmacological inhibition, β2-AR-deficient ADRB2^−/−^ mice exhibited reduced number of lung foci when housed under EE conditions compared to SE housed ADRB2^−/−^ mice (Fig. 3D). Therefore, we concluded that the impact of environmental signals on lung metastasis is independent of adrenergic receptors signaling.

### The impact of environmental signals on lung metastasis is dependent on GR signaling in myeloid cells

**Fig. 4:**
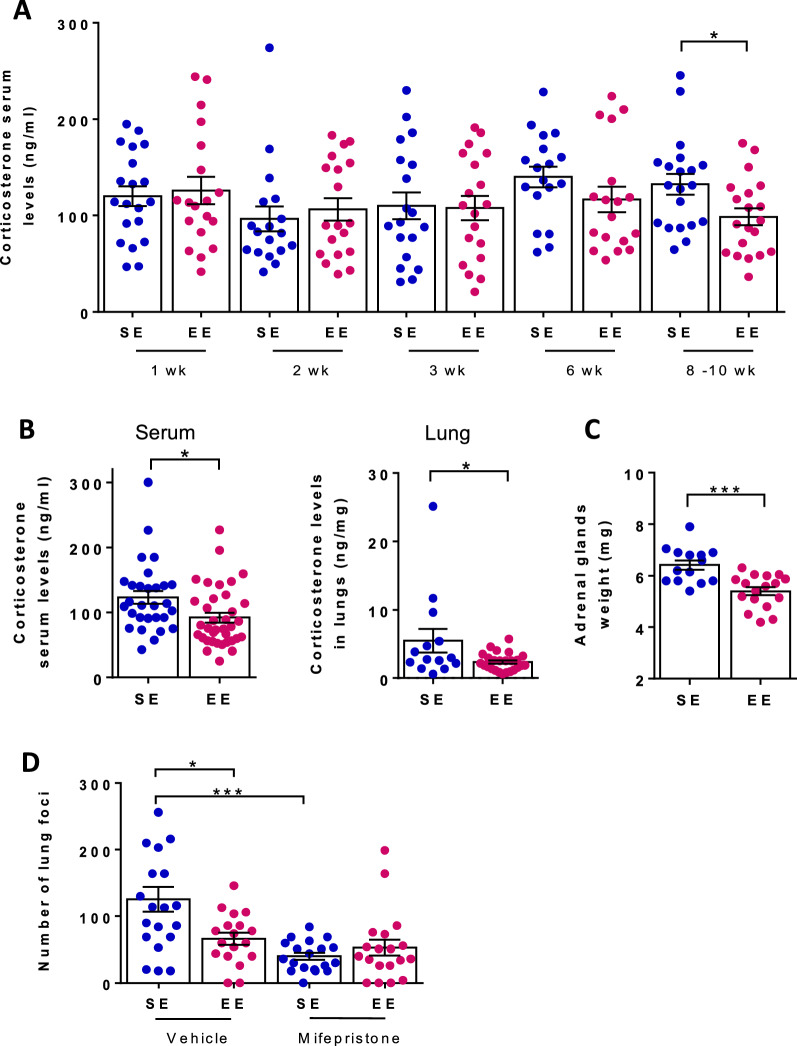
The impact of environmental signals on lung metastasis is mediated by GR-signaling. Wt mice were housed under SE or EE conditions for either the indicated time (**A**) or 10–12 weeks (**B**–**D**). **A** Serum corticosterone levels in individual mice (n = 18–20 for SE, n = 19–21 for EE). **B** Mice were injected with B16:F10 cells into the tail vein and corticosterone levels were measured 2 weeks later in serum (left, n = 30 for SE, n = 40 for EE) and lungs (right, n = 13 for SE, n = 32 for EE). Serum and lung corticosterone levels in individual mice. **C** Adrenal gland weight in individual mice (n = 14 for SE, n = 17 for EE). **D** Mice were treated with either mifepristone (MIFE) or vehicle and injected with B16:F10 cells into the tail vein. Number of lung foci normalized to SE mean in individual mice two weeks after tumor cell injection (n = 18–19 for SE and EE). Mean ± s.e.m. of two (**A**, **D**) or three (**B**, **C**) experiments. *p < 0.05; ***p < 0.001

Because myeloid cells interfere with the metastatic cascade [2, 31–33], and particularly they are the principal immune cells acting during the initial seeding of metastatic cells [33], we investigated whether the impact of environment on lung metastasis was mediated by GR signaling in these cells. As a first step to address the role of GCs, we measured corticosterone levels in SE and EE mice. Whereas serum corticosterone levels were comparable in both groups during the first 6 weeks of specified housing, these were lower in EE mice at 8–10 weeks before (Fig. 4A) and 2 weeks after (Fig. 4B, left panel) injection of B16:F10 cells. Lung corticosterone levels were also lower in EE mice 2 weeks after tumor cell injection (5.5 ± 1.7 ng/mg in SE mice versus 2.4 ± 0.3 pg/ml in EE mice) (Fig. 4B, right panel). Consistent with these results, adrenal glands were smaller (Fig. 4C). Most importantly, blocking GR signaling with the antagonist mifepristone reduced the number of lung metastatic foci in SE mice, but not in EE mice (Fig. 4D). As a result, SE and EE mice exhibited the same number of lung foci when treated with mifepristone indicating that the impact of environment on metastasis was mediated by GR signaling. Since mifepristone is also an antagonist of the progesterone receptor (PR) a more specific strategy to block GR signaling was required.

**Fig. 5:**
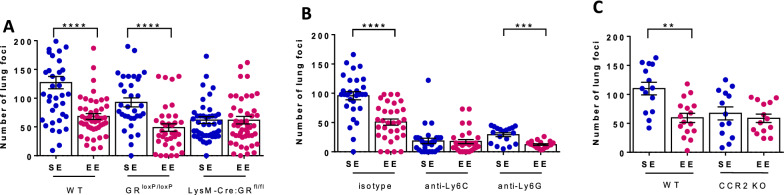
The impact of environmental signals on lung metastasis requires GR-signaling in inflammatory monocytes. Wt (**A–C**), LysM-Cre^+^:GR^fl/fl^ and GR^loxP/loxP^ (**A**, n = 31–55 for SE, n = 32–49 for EE), and CCR2 KO (**C**, n = 13–14 for SE, n = 14–16 for EE) mice were housed under SE or EE conditions for 10–12 weeks and injected with B16:F10 cells into the tail vein. **B** Mice were injected with anti-Ly6C (to deplete inflammatory monocytes), anti-Ly6G (to deplete neutrophils) or an isotype control antibody (n = 30–32 for SE, n = 32–34 for EE). **A**–**C** Number of lung foci normalized to SE mean in individual mice two weeks after tumor cell injection. Mean ± s.e.m. of two (**C**) or three (**A**, **B**) experiments. **p < 0.01; ***p < 0.001; ****p < 0.0001

We used LysM-Cre^+^:GR^fl/fl^ mice [34] in which macrophages, monocytes, neutrophils, and some DCs are selectively deficient in GR signaling (Additional file 1: Fig. S4A and B). Both the frequency and surface phenotype of the main immune cell types were similar in LysM-Cre^+^:GR^fl/fl^ and their control littermates GR^loxP/loxP^ mice (Additional file 1: Fig. S4C). Furthermore both LysM-Cre^+^:GR^fl/fl^ and GR^loxP/loxP^ mice experienced EE-induced behavioral changes, as a measure of changes in the brain. They exhibited decreased anxiety and increased exploratory activity when housed under EE conditions (Additional file 1: Fig. S5), as observed in WT mice. As expected, control littermates GR^loxP/loxP^ mice in EE housing conditions exhibited fewer lung foci than corresponding SE mice (Fig. 5A). In contrast, in SE conditions, the number of lung foci was reduced in LysM-Cre^+^:GR^fl/fl^ compared to GR^loxP/loxP^ mice. As a result, the impact of housing conditions on the difference of number of lung foci between SE and EE was abolished in LysM-Cre^+^:GR^fl/fl^ mice further demonstrating that it is mediated by GR signaling in one or several myeloid cell types.

In agreement with the known pro-metastatic role of these cells, selective depletion of either Ly6G^+^ cells (neutrophils) or Ly6C^+^ cells (iMonos) (Fig. 5B) resulted in reduced number of lung metastatic foci in both SE and EE mice (95.3 ± 7.2 for isotype control versus 29.1 ± 3.2 in SE for anti-Ly6G^+^ and versus 18.6 ± 4.4 in SE for anti-Ly6C^+^; p > 0.0001). Most importantly, while the number of lung foci remained lower in EE mice relative to SE mice following neutrophil depletion, such a difference was abolished by the depletion of Ly6C^+^ cells (Fig. 5B) suggesting that iMonos had a role in the protection of EE mice against lung metastasis seeding. Lungs FACS phenotyping revealed that most Ly6C^+^ cells are CD11c^−^ MHCII^−^ CD24^−^ CD103^−^ and CD11b^high^ indicative of iMonos (Additional file 1: Figs. S6 and S7). In addition, the differences observed between EE and SE in blood inflammatory cytokines (Additional file 1: Fig. S8A) were lost after depletion of Ly6C + cells (Additional file 1: Fig. S8B). To confirm the critical role of these cells in mediating the impact of environmental signals on lung metastasis, we used CCR2 KO mice in which iMonos do not egress from the bone marrow in response to CCL2. We found that CCR2-deficient mice exhibited a similar number of lung foci when housed under SE and EE conditions (Fig. 5D) revealing that CCR2 expression is relevant in this model.

Altogether, our results showed that the impact of environmental signals on lung metastasis is dependent on the presence of an intact inflammatory monocyte compartment.

### Decreased GR signaling in inflammatory monocytes results in an exacerbated inflammatory profile

Inflammatory monocytes are rapidly activated in the lungs as the result of capture of CTC-derived material [33]. To visualize these cells, we injected CMRA-labelled B16:F10 cells into SE and EE mice and analyzed CD45^+^ immune lung cells by flow cytometry 6 h later. CMRA^+^ Ly6C^high^ cells were readily detected in both SE and EE mice (Additional file 1: Fig. S9A). These cells exhibited red cytoplasmic vesicles (Additional file 1: Fig. S9B) confirming that they had captured B16:F10-derived material. However, the frequency and the number of CMRA^+^ Ly6C^high^ were comparable in SE and EE mice (Additional file 1: Fig. S9C). The proportions of CMRA^+^ cells among neutrophils, patrolling monocytes, interstitial macrophages and CD11b^+^ DCs were also similar in both groups (Additional file 1: Fig. S10).

**Fig. 6:**
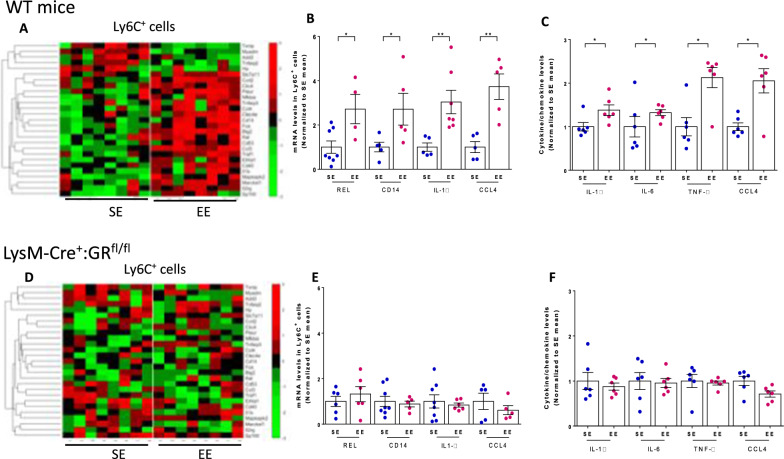
Transcriptomic, cytokine and chemokine profiles of inflammatory monocytes in SE and EE mice. Wt (**A–C**), LysM-Cre^+^:GR^fl/fl^ (**D–F**) mice were housed under SE or EE conditions for 8–10 weeks, injected with B16:F10 cells into the tail vein, and lung Ly6C^+^ cells were sorted 4 days later. (**A**, **D** Lung Ly6C^+^ cells were analyzed by RNA-seq transcriptomic profiling. Clustering heat maps of 8 samples based on the 27 CMRA signature genes that are differentially expressed between SE and EE mice (see Additional file 1: Fig. S11). (BE, n = 5–9 for SE, n = 4–7 for EE) Expression levels of selected genes in Ly6C^+^ cells purified from SE and EE individual mice as determined by quantitative PCR. (**C**, **F**, n = 6 for SE and EE) Lung Ly6C^+^ cells were stimulated with LPS and cellular supernatants were analyzed for the indicated cytokines 16 h later. Cytokine levels in individual mice normalized to the mean levels determined in SE mice. Mean ± s.e.m. of 4–8 mice (**B**, **C**); * p < 0.05; **p < 0.01

Quantitative transcriptome analysis of Ly6C^+^ cells using RNA-seq identified 289 mRNAs (among a total of 4704), that were differentially expressed between CMRA^+^ and CMRA^−^ cells in both SE and EE mice (Additional file 1: Fig. S11A and B; Table S1). Within this “CMRA signature”, many transcripts coded for cytokines, chemokines, and proteins involved in diapedesis and phagocytosis (Additional file 1: Table S2). However, at this early time, no gene was differentially expressed between SE and EE mice neither among CMRA^+^ cells, nor among CMRA^−^ cells (Additional file 1: Fig. S11C). To investigate possible phenotypic differences between SE and EE mice at later time points, we analyzed Ly6C^+^ cells 4 days after B16:F10 cell injection. Among the 289 genes defining the CMRA signature, 27 including those of the transcription factors fos and rel, the cytokines IL-1ß, the chemokines CCL3 and CCL4, and the LPS coreceptor CD14, were expressed at higher levels in EE mice (Fig. 6A). Similar differences were found by quantitative PCR in independent experiments (Fig. 6B).

To further characterize Ly6C^+^ cells from SE and EE mice, we purified these cells from the lung of tumor cell-injected mice and stimulated them with LPS in vitro as a TLR4 ligand. It has been shown that GR signaling affects TLR4 signaling cascade in the myeloid compartment (40). We found that Ly6C^+^ cells from EE mice produced more IL-1β, IL-6, TNF-α and CCL4 than corresponding cells from SE mice when stimulated with LPS (Fig. 6C), confirming that these cells were functionally distinct. We then investigated whether these phenotypic differences between SE and EE Ly6C^+^ cells were abolished when the GR was absent in myeloid cells. In contrast to what occurred in wt mice, Ly6C^+^ cells from LysM-Cre^+^:GR^fl/fl^ mice exhibited similar transcriptomic profiles in SE and EE housing conditions (Fig. 6D and E, Additional file 1: Fig. S12). Likewise, Ly6C^+^ cells from LysM-Cre^+^:GR^fl/fl^ mice secreted similar cytokine levels upon LPS stimulation in SE and EE mice (Fig. 6F).

Altogether, our results show that Ly6C^+^ cells from SE and EE mice were phenotypically different 4 days after tumor cell injection, with those from EE mice exhibiting an exacerbated inflammatory profile. These phenotypic differences were abolished in LysM-Cre^+^:GR^fl/fl^ mice further supporting the role of GR signaling in this phenomenon.

### Relationship between the increased inflammation and the reduced number of lung metastatic foci

**Fig. 7:**
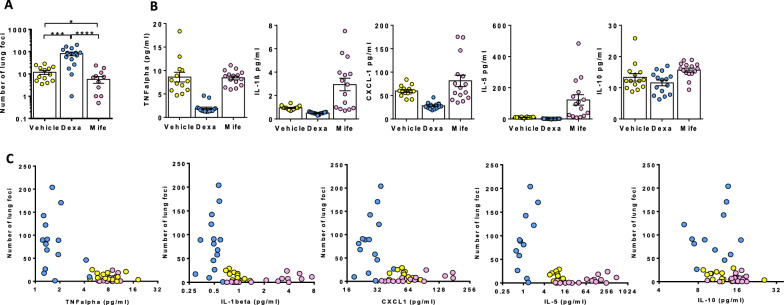
Impact of GR agonists and antagonists on lung foci and serum cytokine levels. SE mice were treated from day − 3 to day 3 with the GR agonist dexamethasone (Dexa), the GR antagonist mifepristone (Mife) or vehicle. 10^4^ B10:F10 tumor cells were injected into the tail vein on day 0. **A** Number of lung foci in individual mice on day 15 (n = 13 for vehicle, n = 14 for Dexa and n = 11 for Mife). (**B**, n = 13–15 for vehicle, n = 15 for Dexa and Mife) Serum levels of TNF-α, IL-1β, CXLC1, IL-5 and IL-10 in individual mice on day 4. **C** Scatter plot representation of lung foci number on day 15 and serum cytokine levels on day 4 in mice treated with mifepristone (pink circles, n = 15), dexamethasone (light blue circles, n = 15) or vehicle (yellow circles n = 13). Mean ± s.e.m. of 12–15 mice. *p < 0.05; **p < 0.01; ***p < 0.001; ****p < 0.0001

While the reduction in the number of lung foci in EE mice was associated with increased inflammation in lungs, it remains to be determined whether there is a causal relationship between these two phenomena. To address this issue, we treated SE mice with either the GR agonist dexamethasone or the GR antagonist mifepristone before and shortly after injection of a limiting dose of B16:F10 cells. As expected with the role of GR in metastasis development, in SE mice the number of lung foci on day 15 was reduced by 2-3-fold in mifepristone-treated mice and increased by 8.4-fold in dexamethasone-treated mice compared to control animals respectively (Fig. 7A). We next asked whether there was an association between the number of lung foci at day 15 and cytokine serum level at day 4 in SE mice. While low levels of TNF-a, IL-1b, CXCL1 and IL-5 were measured in the blood, these levels were significantly reduced in dexamethasone-treated mice relative to control animals four days after injection (Fig. 7B). In contrast, treatment with mifepristone resulted in increased serum levels of both IL-1β and IL-5. When mice from all groups were analyzed together, the number of lung foci on day 15 decreased with serum levels of TNF-α, IL-1β, CXCL1 and IL-5 on day 4, but not with those of IL-10 (Fig. 7C). More specifically, mice exhibiting cytokine levels above a specific threshold, i.e. 4 pg/ml of TNF-α, 0.6 pg/ml of IL-1β, 32 pg/ml of CXCL1, 3.0 pg/ml of IL-5, had less than 50 lung metastatic foci, suggesting a cytokine threshold-based mechanism of foci development.

Altogether, these data strongly suggest that there is a causal relationship between the increased inflammation that is observed in the lungs of EE mice and the reduced number of lung metastatic foci found in these animals.

## Discussion

In several malignancies, bone marrow derived IMs are recruited to the tumor microenvironment by tumor cells, where they become metastasis associated macrophages (MAMs) and have been shown to play key roles in the metastatic process [35]. As instance, IMs have been shown to be preferentially recruited early to metastatic sites, such as the lung and liver, via production of the monocyte chemoattractant cytokines [36, 37]. IMs and MAMs also play a role in promoting metastatic tumor cell extravasation and growth at sites of metastasis [36, 38, 39]. These results in animal models are strengthen by clinical studies demonstrating a negative prognostic role for increased numbers of IMs and elevated serum CCL2 concentrations in patients with various malignancies [40–43] and suppression of tumor growth in patients with bone metastases by blockade of the CCL2 receptor [44]. Consistently, we found that the absence of CCR2 expression was beneficial when mice were housed in SE, resulting in a decrease of metastatic foci.

In this study, we have found here that IMs showed both increase inflammatory signature at an early stage of the metastatic process and anti-tumor properties in EE housing conditions. These results demonstrate that IMs can be reprogrammed by brain-derived signals to inhibit metastasis development. While we did not further investigated the mechanism relating the causal relationship between the increased inflammation and the reduced number of lung metastatic foci, it is noteworthy that for the two metastasis models tested, EE mice exhibited increased numbers of T-cells and NK cells. Because both these cell types inhibit lung metastasis [45, 46], and although we do not rule out an important role for other cell types including inflammatory monocytes themselves [47], our data support a model in which increased levels of chemokines and inflammatory cytokines in EE mice promote the migration and/or the lytic activity of CD8^+^ T-cells and NK cells in lungs (Additional file 1: Fig. S13). In agreement with the increased numbers of lymphocytes and NK cells, the lungs of EE mice also contained higher levels of CCL20, CXCL2 and CXCL10 that are all chemotactic for these cells.

Most studies aimed at investigating the impact of neural signals on the immune system have used “negative” stress models, such as inescapable foot shocks, maternal separation, physical constraint or social defeat. In contrast, EE mice are exposed to “positive” stress, a concept first proposed by Hans Selye to explain how environmental signals perceived by the brain could induce adaptive physiological changes with beneficial effects on health [48]. Many authors have reported that EE mice exhibited smaller primary tumors compared to control SE mice which is, at least partially attributed to increase SNS activity [21, 22, 49–53]. However, none of these authors have explored the impact of EE on tumor metastasis. Here we show that not only EE housing conditions decreased the number of lung metastasis in mice compared to SE housing conditions but also that this protection against metastasis was not dependent on ARs signaling but rather on GR signaling.

The impact of GCs on immune cell function has been extensively studied. For example, in vitro experiments have shown that GCs could induce the apoptosis of immature dendritic cells (DCs), increase antigen uptake by mature DCs and promote the development of tolerogenic DCs by inhibiting the expression of MHC-II, co-stimulatory molecules, and pro-inflammatory cytokines [54]. Further, low and high dose of GCs promote and inhibit the production of pro-inflammatory cytokines by macrophages respectively [55, 56]. GCs can also drive the differentiation of monocytes toward a specific anti-inflammatory subtype, which can migrate rapidly into inflamed tissues to help resolve inflammation [57, 58]. Other authors have investigated the impact of endogenous GCs on myeloid cells in vivo. For example, GR signaling in myeloid cells was found to be beneficial in acute Graft Versus Host Disease [59], Dextran Sodium Sulfate-induced colitis [60], contact hypersensitivity [34] and septic shock [61]. In agreement with the ability of corticosterone to inhibit inflammation, we have found here that myeloid cells exhibited an altered transcriptomic profile and secreted more pro-inflammatory cytokine in EE mice that exhibit lower levels of endogeneous GCs. These differences were observed 3 and 4 days after B16:F10 cell injection, but neither before nor 6 h after CTCs have reached the lung. While GR is constitutively expressed and controls many distinct gene networks, the specificity of GR-mediated responses depends on combinatorial, context-specific assembly of GR-nucleated transcription regulatory complexes at genomic response elements [62]. Further studies should be performed to elucidate when and which of these complexes are solicited when inflammatory monocytes are activated by CTCs.

GC production by adrenal glands is regulated by the adrenocorticotropic hormone (ACTH), which is secreted by the anterior pituitary gland in response to corticotropin-releasing hormone (CRH) produced in the hypothalamus. GCs exert negative feedback on both ACTH and CRH. Because the hypothalamus not only regulates CRH production, but also integrates sensory signals including environmental stimuli, it is likely to be responsible for the difference in GCs set point between SE and EE mice. The molecular mechanisms that are responsible for this phenomenon remain to be elucidated.

Because the social and physical environment in which we live has a profound impact on cancer incidence or progression [5], it is interesting to speculate about the relevance of this study for humans. Positive factors such as social support and optimism predict longer survival [9, 10]. Also, cortisol output has been consistently shown to be lower among individuals reporting positive affect [63]. However, to the best of our knowledge, there is currently no study in which psychosocial wellbeing, cortisol output and cancer progression were assessed altogether. Such a study would be warranted to help elucidating the molecular mechanisms by which positive environmental factors impact cancer progression.

GCs are routinely administered in cancer chemotherapy to mitigate untoward inflammation. An unexpected finding of this study is that SE mice treated with the GR agonist dexamethasone exhibit not only reduced serum levels of pro-inflammatory cytokines 4 days after tumor cell injection, but also increased number of lung metastatic foci on day 15. In striking contrast, SE mice treated with the GR antagonist mifepristone exhibit both increased serum levels of pro-inflammatory cytokines and reduced number of lung metastatic foci. These findings raise questions regarding the near systematic use of corticoids during cancer treatment. Most studies in animal models show that glucocorticoids administration increase metastatic colonization and reduces survival in breast cancer mouse model [64]. While some retrospective clinical studies have shown that co-administration of glucocorticoids in patients with lung or breast cancer increases metastasis [65], prospective clinical trials dedicated to assess the effect of glucocorticoids on metastasis are necessary.

In addition to the potential of glucocorticoids to render tumor cells resistant to apoptosis [65] or to increase intra-patient tumor heterogeneity [64], our study shows that GR signaling in myeloid cells could promote lung metastasis. Therefore, suggesting that caution is needed when using glucocorticoids to treat patients at risk of developing lung metastasis.

## Materials and methods

### Mice, animal housing and treatments

All experiments were performed with female C57BL/6 mice (Charles River). ADRB2^−/−^ [66], GR^loxP/loxP^ [67] and LysM-Cre [68] mice were described before. Mice were housed as previously described [69]. For treatment with AR antagonists, 3 wk-old mice were assigned to live in SE or EE and either propranolol or phenoxybenzamine were provided in drinking water at 0.5 g/l and 0.25 g/l respectively. For treatment with GR antagonists or agonists, mice were injected i.p. with either mifepristone (80 mg/kg in 10% DMSO), or dexamethasone (5 mg/kg in 10% DMSO), or vehicle alone daily starting 2 days before tumor cell injection during 5 days. For antibody-mediated cell depletion, mice were injected i.p. with either anti-Ly6C (Monts-1), or anti-Ly6G (1A8) or control isotype mAb. Antibodies were injected 1 day before tumor cell injection (500 µg/mouse), as well as on day 0, 1, 4, 8 and 12 (200 µg/mouse).

### Behavioral tests and hippocampal neurogenesis assay

All behavioral testing occurred during the light phase. For the rotarod test, mice were placed on a rotating wheel for two consecutive 5 min-habituation phases on fixed rod (4 rpm/min) four hours apart. Twenty-four hours later, the latency to fall was recorded on accelerating rod (from 4 to 40 rpm/min). The latency to fall was used as a measure for motor coordination.

For the open-field (OF) test, mice were placed in the corner of the test apparatus (45 cm long, 45 cm width, 25 cm height Plexiglas box) and activity was recorded for 10 min. The time spent in the aversive centre of the apparatus is inversely correlated to the anxiety-like behavior.

For the light-dark (L&D) preference test, the test apparatus (40 cm long, 30 cm width, 25 cm height Plexiglas box) was divided into equal zones (i.e., light or dark zones) with a doorway connecting the two sides. The light zone was very bright (200 lux) while the dark zone was protected from light by an opaque lid. To initiate testing, mice were placed into the light side and activity was recorded for 5 min. The latency of first entry in the dark zone and the time spent in the light area are reliable parameters to assess the anxiety-like behavior of mice.

For the Forced Swim Test (FST), mice were placed into glass buckets (20 cm diameter, 30 cm deep, filled with 22 °C ± 0.5 °C water) and videotaped for the entire session. As already described (7), only the last 4 min were scored for immobility duration. A mouse was considered immobile when it remained floating in an upright position with only slight movements to keep its head above water.

For the Novelty Suppressed Feeding (NSF) test, the testing apparatus consisted of a plastic box, the floor of which was covered with approximately 2 cm of wooden bedding. Twenty hours prior to testing, mice were subjected to fasting in their home cage. At the time of testing, a single pellet of food was placed on a Plexiglass platform in the center of the box. An animal was placed in a corner of the box, and the entire session was videotaped (i.e. 10 min. period). The latency to eat (defined as the mouse sitting on its haunches and biting the pellet) was timed.

For the sociability and social novelty preference tests, the testing apparatus was a rectangular, three-chambered box with clear Plexiglas walls, having small circular openings (3.5 cm in diameter) allowing access into each chamber (25 cm long, 15 cm width, 20 cm height). Three interconnected chambers are separated by manually operated sliding doors. The left and right chambers contained a see-through circular box perforated with multiple holes (0.5 cm diameter) for receiving stranger mice. The test mouse was first placed in the middle chamber and allowed to explore the entire apparatus for five minutes. During the habituation period, the perforated boxes were empty. At the end of the session, the test mouse was confined into the central chamber by obstruction of the doorways between the two side chambers. An unfamiliar mouse (same genotype, sex and age than the tested mouse) was enclosed in the perforated box of one of the side chambers. Both doors to the side chambers were then unblocked and the test mouse was allowed to explore the entire social test box for a 5-minute session. The test was videotaped, and the time spent in each chamber of the apparatus during the two 5-minute sessions was measured.

### Cell lines and cell culture

B16:F10 melanoma and Lewis lung carcinoma (LLC) LL2 cells were obtained from the American Type Culture Collection (ATCC).

To produce GFP-expressing B16:F10 cells, B16:F10 cells were transduced with a lentivirus obtained from cells transfected with the eukaryotic expression vector pRRLSIN.cPPT.PGK-GFP.WPRE carrying the GFP gene (Addgene plasmid 12,252, gift from D. Trono). GFP^+^ cells were sorted by flow cytometry, cloned, and expanded.

When indicated, B16:F10 cells were incubated with 20 µM CMRA (Molecular Probes) in complete medium for 30 min at 37 °C. Cells were washed in twice PBS 1X prior to injection into mice.

For LPS stimulation, Ly6C^+^ cells were sorted by flow cytometry and incubated in flat bottom 96-well plates (1000 cells per well) with or without 150 ng/ml of LPS for 16 h in DMEM supplemented with 10% Fetal Bovine Serum, 1 mM sodium pyruvate, 4 mM L-glutamine and 50 µM 2-mercaptoethanol in 5% CO_2_ at 37 °C.

For assessing response to corticosterone, peritoneal macrophages were counted and incubated for 1 h in flat bottom 96-well plates (10^5^ cells per well) in DMEM supplemented with 10% Foetal Bovine Serum, 1 mM sodium pyruvate, 4 mM L-glutamine and 50 µM 2-mercaptoethanol in 5% CO_2_ at 37 °C. Non-adherent cells were discarded and adherent cells were incubated for 16 h in complete medium with 150 ng/ml of LPS and various concentrations of corticosterone.

### Liver and lung metastasis models

In the liver metastasis model, 10^5^ LL2 cells in 100 µl of PBS were injected into the portal vein and mice were sacrificed 21 days later. Liver tumors were dissected out and weighted.

In the lung metastasis model, 10^5^ B16.F10 melanoma cells in 100 µl of PBS were injected into the tail vein and mice were sacrificed 14 days later (except for Fig. 7, for which only 10^4^ B16.F10 melanoma cells were injected). Lungs were dissected out, separated into their five lobules and macroscopic metastatic foci were counted under binoculars in a blinded fashion by three different investigators. Results are presented as an average of the three countings.

### Antibodies

BrdU, mifepristone and corticosterone were purchased from Sigma. CMRA was purchased from Molecular Probes. DAPI and 7-AAD were purchased from BD Bioscience.

For flow cytometry experiments, Anti-CD3 (17A2), anti-CD4 (GK1.5), anti-CD8 (53 − 6.7), anti-CD11b (M1/70), anti-CD19 (1D3), anti-CD24 (M1/69), anti-CD45 (30-F11) anti-CD64 (X54-5/7.1), anti-CD45 (30-F11), anti-F4/80 (CI:A3-1), anti-Ly6C/G (RB6-8C5), anti-Ly6C (AL-21), anti-Ly6G (1-A8), anti-NKp46 (29A1.4), anti-Siglec-F (E50-2440) mAb were purchased from BD Bioscience. Anti-CD11c (N418), anti-CD103 (2E7) and anti-MHCII (M5/114) mAb were purchased from e-Biosciences. For antibody-mediated cell depletion experiments, anti-Ly6C/G (RB6-8C5), anti-Ly6C (Monts-1) and anti-Ly6G (1-A8) were purchased from BioXCell.

### Chemokine, cytokine, corticosterone and noradrenaline levels

Chemokine and cytokine levels were measured using an Electro-Chemo-Luminescence (ECL)-based assay (v-plex®, MesoScaleDiscovery). Noradrenaline levels were measured by ELISA (DLD Diagnostika) following manufacturer instructions. For corticosterone levels, blood samples were collected by retro-orbital collection 1–2 h before lights were switched off. Blood was drawn within 3 min after opening the grid with minimal manipulation prior to sampling and corticosterone was measured by ELISA kit (Enzo Life Sciences).

### Single-cell suspension and purification

Lungs were minced in small pieces and enzymatically dissociated in HBSS Ca^2+^ Mg^2+^ containing 0.2 mg/ml DNase I (Roche), 0.1 mg/ml Liberase®, 1 mg/ml hyaluronidase, 0.25 mg/ml collagenase IX (Worthington) during 45 min. Cellular suspensions were homogenized by serial pipetting and passed through a nylon cell strainer (Becton Dickinson) in 5% (vol/vol) FCS and 2.5 mM EDTA (Sigma-Aldrich) in HBSS. For sorting Ly6C^+^ cells, lung cells were stained with anti-CD45, anti-CD11b, anti-Ly6C anti-Ly6G mAb and DAPI. CD45^+^ CD11b^+^ Ly6C^+^ Ly6G^−^ DAPI^−^ were sorted by flow cytometry using a BD FACSAria III (BD Biosciences).

### Flow cytometry analysis

Alveolar macrophages (CD11b^low^ CD11c^+^ CD64^+^ Ly6C^−^ Ly6G^−^ Siglec-F^+^), interstitial macrophages (CD11b^+^ CD11c^+^ CD24^−^ Ly6C^+^ Ly6G^−^ MHCII^+^ Siglec-F^−^), patrolling monocytes (CD11b^+^ CD11c^med^ CD24^−^ Ly6C^low^ Ly6G^−^ MHCII^−^ Siglec-F^−^), inflammatory monocytes (CD11b^+^ CD11c^−^ CD24^−^ Ly6C^high^ Ly6G^−^ MHCII^+/−^), CD11b^+^ DCs (CD11b^+^ CD11c^+^ CD24^+^ CD103^−^ MHCII^high^ Ly6C^−^ Ly6G^−^), CD103^+^ DCs (CD11b^−^ CD11c^+^ CD24^+^ CD103^+^ MHCII^high^ Ly6C^−^ Ly6G^−^), neutrophils (CD11b^+^ CD11c^−^ F4/80^−^ Ly6C^−^ Ly6G^high^), eosinophils (CD11b^high^ CD11c^−^ F4/80^int^ Ly6C^int^ Ly6G^int^ Siglec-F^+^), CD4^+^ lymphocytes (CD4^+^ CD3^+^), CD8^+^ lymphocytes (CD8^+^ CD3^+^), B lymphocytes (CD19^+^ CD3^−^) and NK cells (CD3^−^ NK1.1^+^) were identified by flow cytometry.

Cytometry was performed on a SP6800®, Spectral Cell Analyzer-Sony Biotechnology or LSRII Fortessa-BD Biosciences using the gating strategy described in fig. S6A. Cells were analyzed using a Spectral Analyser and the Kaluza® Flow Analysis Software after gating on live cells.

### Quantitative PCR

Lungs were mechanically dissociated using Lysing Matrix D tubes (FastPrep®, MP biomedical) and RNA were isolated using the miRNEasy® micro kit, Quiagen) following manufacturer instructions. RNAs were quantified by Nanodrop similar amounts of RNA were used to perform RT-PCR using the QuantiTect® Reverse transcription kit (Quiagen) and the SyberGreen® Master Kit (Roche). Amplicons were quantified using a LightCycler 480 II (Roche). Primers were designed according to PrimerBank (Harvard University). mRNA cytokine levels were normalized to GAPDH using LightCycler software (Roche).

### RNA-seq transcriptomic profiling

RNA-seq transcriptomic profiling was performed as previously described [70]. Differential expression and signature analysis were performed using Bioconductor packages (http://www.bioconductor.org/). Integration of all data was performed with the support of Mediante tools and data mining tools such as Ingenuity Pathway®.

### Statistical analysis

Student’s t-test and Mann-Whitney-Wilcoxon test were performed to assess statistical significance of Gaussian and non-Gaussian distributed data respectively.

## Supplementary Information

**Additional file 1: Fig. S1.** Behavior and adult hippocampal neurogenesis in SE and EE mice.SE and EE mice were analyzed using the openfield test (A), the light and dark paradigm test (B), the Novelty SuppressedFeeding (NSF) test, (C) the Forced Swimming Test (FST) (D), and the Barnes mazetest (E). (A) Time spent in the aversive center of the open field arena inseconds (left). Number of entries in the central area (right). (B) Time spentin light. (C) Latency to eat. (D) Immobility time. (E) Spatial learning curveestablished by training in the Barnes maze for four consecutive days. (F) Numberof BrdU-positive nuclei in the dentate gyrus three weeks after BrdU treatment.Each dot represents a mouse (A-D, F). Mean ± s.e.m. of two experiments. *, p < 0.05; **, p < 0.01; ****, p < 0.0001. **Fig. S2.** Number of lymphoid and myeloid immune cell types in thesecondary lymphoid organs of SE and EE mice before tumor cell injection.C57BL/7 mice were housed under SE or EEconditions for 10-12 weeks and cell suspensions were prepared from the spleen(A, B) and mesenteric LN (C). (A) CD4^+^ T lymphocytes (CD4^+^CD3^+^), CD8^+^ T lymphocytes (CD8^+^ CD3^+^),B lymphocytes (CD19^+^ CD3^-^), NK T cells (NK1.1^+^CD3^+^), NK cells (NK1.1^+^ CD3^-^), neutrophils(CD11b^+^ CD11c^-^ F4/80^-^Ly6C^-^ Ly6G^high^),CD11b^+^ DCs (CD11b^+^CD11c^+^), CD8^+^DCs (CD8^+^CD11c^+^), plasmacytoid DCs (CD11c^+^CD11b^-^120G8^+^),naïve CD4^+^ T cells (CD4^+^ CD3^+^ CD44^low^,CD62L^high^), effector/memory CD4^+^ T cells (CD4^+^CD3^+^ CD44^high^) , regulatory CD4^+^ T cells (CD4^+^FoxP3^+^), naive CD8^+^ T cells (CD8^+^ CD3^+^CD44^low^, CD62L^high^), memory CD8^+^ T cells (CD8^+^CD3^+^ CD44^high^) were identified by flow cytometry based onthe indicated surface markers. (A) Number of lymphoid cell types in spleennormalized to the mean values determined in SE mice. (B) Number of myeloid celltypes in spleen normalized to the mean values determined in SE mice. (C) Numberof lymphoid cell types in mesenteric LN normalized to the mean valuesdetermined in SE mice. Mean ±s.e.m. of two experiments. **Fig. S3.** Cytokine and chemokine levels and frequency of immune celltypes in the lungs of SE and EE mice before tumor cell injection.C57BL/7 mice were housed under SE or EEconditions for 10-12 weeks. (A, B) Lung protein extracts were prepared and thelevels of the indicated cytokines (A) and chemokines (B) were measured. (C, D)Lung cell suspensions were prepared and the number of NK cells (NK1.1^+^CD3^-^), T lymphocytes (CD19^-^ CD3^+^), Blymphocytes (CD19^+^ CD3^-^), inflammatory monocytes(Siglec-F^-^CD11c^-^ Ly6G^-^CD11b^high^MHCII^-^ CD64^-^ Ly6C^+^), patrolling monocytes(Siglec-F^-^CD11c^-^ Ly6G^-^CD11b^high^MHCII^-^CD64^-^Ly6C^-^), neutrophils (Siglec-F^-^CD11c^-^ CD103^-^ CD11b^+^Ly6G^high^),CD11b^+^ DCs (Siglec-F^-^CD11c^+^ CD11b^high^MHCII^+^CD64^-^ CD24^+^), CD103^+^ DCs(Siglec-F^-^CD11b^-^CD103^+^ CD11c^+^CD24^+^),interstitial macrophages (Siglec-F^-^CD11c^-^ CD11b^high^MHCII^+^CD64^+^ CD24^-^), alveolar macrophages(CD11b^-^ Siglec-F^+^CD11c^+^ CD64^+^) wasdetermined by flow cytometry based on the indicated surface markers. (A)Cytokine levels in lung normalized to the mean values determined in SE mice.(B) Chemokine levels in lung normalized to the mean values determined in SEmice. (C) Number of myeloid cell types in lung normalized to the mean valuesdetermined in SE mice. (D) Number of lymphoid cell types in lung normalized tothe mean values determined in SE mice. Mean ± s.e.m. of two experiments. **Fig. S4.** Phenotypiccharacterization of LysM-Cre^+^:GR^fl/fl ^and LysM-Cre^+^: Stop^fl/+^TdTomato  transgenic mice.    (A) Lungcells from LysM-Cre^+^:Stop^fl/+^TdTomato micewere analyzed by flow cytometry after gating on CD45^+^ cells and thefrequencies of TdTomato^+^ cells among alveolar macrophages (CD11b^low^CD11c^+^ CD64^+^ Ly6C^-^Ly6G^-^ Siglec-F^+^),interstitial macrophages (CD11b^+^ CD11c^+^ CD24^-^Ly6C^+^ Ly6G^-^ MHCII^+^Siglec-F^-^),neutrophils (CD11b^+^ CD11c^-^ F4/80^-^Ly6C^-^Ly6G^high^), inflammatory monocytes (CD11b^+^ CD11c^-^CD24^-^Ly6C^high^ Ly6G^-^MHCII^+/-^) patrollingmonocytes (CD11b^+^CD11c^med^ CD24^-^ Ly6C^low^Ly6G^-^ MHCII^-^ Siglec-F^-^), DCs (CD11c^+^Ly6C^-^Ly6G^-^), and T lymphocytes (CD3^+^), were determined.Frequency of TdTomato^+^cells among the indicated cell types in arepresentative mouse. (B) Peritoneal macrophages from LysM-Cre^+^:GR^fl/fl^andGR^loxP/loxP^ mice were incubated with LPS in the presence ofthe indicated concentrations of corticosterone. Cellular supernatants wereassessed for TNF-a secretion 24 hours later. Percentage ofinhibition of TNF-a secretion relative to cells incubated in theabsence of corticosterone. Mean ± s.e.m. of 4 mice/group. (C) Lung, spleen andblood cells from LysM-Cre^+^:Stop^fl/+^TdTomato and GR^loxP/loxP^. mice were analyzed by flow cytometry aftergating on CD45^+^ cells for all immune cells and CD45^-^cells for endothelial cells. Mean ±s.e.m. of 4 mice/group. **Fig. S5.** Behavioralcharacterization of LysM-Cre^+^:GR^fl/fl^ .(A-D) GR^loxP/loxP^  and LysM-Cre^+^:GR^fl/fl ^micewere housed for 10 weeks under SE or EE conditions and analyzed using the openfield test (A), the light and dark paradigm test (B), the Novelty SuppressedFeeding (NSF) test (C) and the forced swimming test (FST) (D). (A) Time spentin the aversive center of the open field arena. (B) Time spent in light. (C)Latency to eat. (D) Immobility time. Mean ±s.e.m.; **, p < 0.01; ***, p < 0.001; ****, p < 0.0001. **Fig. S6.** Flow cytometry gating strategy used for analyzinglung-infiltrating immune cells and immunophenotyping of Ly6C-depletedmice. (A) Flowcytometry gating strategy. After isolation, lung cells were stained with 7-AADand mAbs to CD11b, CD11c, CD24, CD45, CD64, CD103, F4/80, Ly6C, Ly6G, MHCII,Siglec-F and analyzed by flow cytometry. The gating strategy for identifyingalveolar macrophages (AM)(CD11b^-^ Siglec-F^+^CD11c^+^CD64^+^), CD103^+^ DCs (Siglec-F^-^CD11b^-^CD103^+^CD11c^+^CD24^+^), neutrophils (Siglec-F^-^CD11c^-^CD103^-^ CD11b^+^ Ly6G^high^), inflammatorymonocytes (iMo) (Siglec-F^-^CD11c^-^ Ly6G^-^CD11b^high^MHCII^-^ CD64^-^ Ly6C^+^), patrolling monocytes(pMo) (Siglec-F^-^CD11c^-^ Ly6G^-^CD11b^high^MHCII^-^ CD64^-^ Ly6C^-^), interstitial macrophages(IM) (Siglec-F^-^CD11c^-^ CD11b^high^ MHCII^+^CD64^+^CD24^-^) and CD11b^+^ DCs (Siglec-F^-^CD11c^+^CD11b^high^ MHCII^+^CD64^-^ CD24^+^) isshown. (B) Lung, slpeen and blood cells from Isotype-control and anti-Ly6C-treated mice were analyzed by flow cytometry aftergating either on CD45^+^ cells for all immune cells or CD45^-^cells for endothelial cells. It is to be noted that anti-Ly6C clone Monts-1 wasused as depleting antibody whereas anti-Ly6C clone AL-21 was used for flowcytometry staining. Mean ± s.e.m. of 6 mice/group. ***, p < 0.001; n.s. notsignificant. **Fig. S7.** Flowcytometry analysis of Ly6C^+^ cells in lung. After isolation, lung cells from wt mice werestained with mAbs to CD103, CD11b, CD11c, CD24, CD45, Ly6C, Ly6G, MHCII andanalyzed by flow cytometry after gating on CD45^+^ Ly6C^+^alive cells. Representative FACS profiles and proportions of cells within the indicatedgates. **Fig. S8.** Cytokineserum levels 4 days after tumor cell injection.C57BL/7 mice were housed under SE or EEconditions for 10-12 weeks. (A) Mice were injected with B16:F10 cells into thetail vein and serum samples were analyzed 4 days later for IFN-g,IL-1b,IL-6, TNF-a, and IL-10 levels. (B) Mice were treated witheither an anti-Ly6C or and isotype control mAb and injected one day after withB16:F10 cells into the tail vein. Serum samples were analyzed 4 days later forIFN-g,IL-1b,IL-6, TNF-a, and IL-10 levels. Cytokine levels inindividual mice normalized to the mean values determined in SE mice. Mean ±s.e.m. of three (A) or two (B) experiments. *, p < 0.05; **, p < 0.01;***, p < 0.001; ****, p < 0.001. **Fig. S9.** Differential transcriptomic, cytokine and chemokine profiles of inflammatorymonocytes in EE and SE mice.SE and EE mice were injected with CMRA-labelledB16:F10 cells and analyzed 6 hours later. (A-C) Lung cells were stained withDAPI and antibodies to CD45, CD11b and Ly6C, and analyzed by flow cytometry (A,C) or confocal microscopy after sorting of CMRA^+^Ly6C^high^cells(B). (A) Gating strategy. (B) Confocal analysis of representative CMRA^+^Ly6C^high^cells purified from SE mice after staining with anti-CD45 mAb (green).Arrows indicate CMRA^+^ cytoplasmic vesicles (red). (C) Number of CMRA^+^inflammatory monocytes in individual SE and EE mice. **Fig. S10.** Percentage of CMRA^+^ cells in SE and EE mice.C57BL/7 mice were housed under SE or EEconditions for 10-12 weeks and injected with CMRA-labeled B16:F10 cells. Lungcell suspensions were prepared 16 hours later and the percentage of CMRA^+^cells was measured after gating on neutrophils (Siglec-F^-^CD11c^-^CD103^-^ CD11b^+^ Ly6G^high^), inflammatorymonocytes (Siglec-F^-^CD11c^-^ Ly6G^-^CD11b^high^MHCII^-^ CD64^-^ Ly6C^+^), patrolling monocytes(Siglec-F^-^CD11c^-^Ly6G^-^CD11b^high^MHCII^-^ CD64^-^Ly6C^-^), interstitial macrophages(Siglec-F^-^CD11c^-^CD11b^high^MHCII^+^CD64^+^CD24^-^) and CD11b^+^ DCs (Siglec-F^-^CD11c^+^CD11b^high^ MHCII^+^CD64^-^ CD24^+^).Percentage of CMRA^+^ in individual mice normalized to the mean valuesdetermined in SE mice. Mean ± s.e.m. two experiments. **Fig. S11.** Differential transcriptomic profiles of inflammatory monocytes in EE and SEmice.SE and EE mice were injected with CMRA-labelledB16:F10 cells and analyzed 6 hours later. Lung cells were stained with DAPI andantibodies to CD45, CD11b and Ly6C. CMRA^+^and CMRA^-^Ly6C^high^cells were sorted and analyzed by RNA sequencing. (A) Volcano plotanalysis of genes differentially expressed in CMRA^+^ and CMRA^-^Ly6C^+^ cells in SE (left) and EE (right) mice. (B) Scatter plotrepresentation showing, for each transcript, the ratio between expression levelin CMRA^+^ and CMRA^-^ cells in EE mice (Y axis) and in SEmice (X axis). (C) Clustering heat map of 16 samples based on the 289 genesdifferentially expressed between CMRA^+^ and CMRA^-^ cells inSE and EE mice 6 hours after tumor cell injection. **Fig. S12.** Transcriptomicanalysis of Ly6C^+^ cells from LysM-Cre^+^:GR^fl/fl^mice.LysM-Cre^+^:GR^fl/fl^miceand GR^loxP/loxP^ were housed for 10-12 weeks under SE or EEconditions, and injected with CMRA-loaded B16:F10 cells into the tail vein.Lung cell suspensions were prepared 4 days later, and Ly6C^+^ cellswere sorted by flow cytometry and analyzed by RNA-seq transcriptomic profiling.Expression levels of CCL3, CCL4, Fos, Rel and IL-1b inindividual mice. Mean ± s.e.m. of 8 mice. *, p < 0.05; **, p <0.01; ***, p < 0.001. **Fig. S13.** Schematic representation of the impact of the housingenvironment on the immune system during the late stages of lung metastasis.(A) Schematic representation of the impact ofenriched environment on the hypothalamic-pituitary-adrenal axis. Sensory,cognitive and motor stimuli are integrated in the hypothalamus. Glucocorticoidproduction by adrenal glands is regulated by the adrenocorticotropic hormone(ACTH), which is secreted by the anterior pituitary gland in response tocorticotropin-releasing hormone (CRH) produced in the hypothalamus. In EE mice,the activity (and weight) of adrenal glands is decreased resulting in lowercorticosterone serum levels. (B, C) Schematic representation of theinteractions between immune and tumor cells in the lung microenvironment 0-6hours (B) and 3-4 days (C) after B16:F10 cell injection. (B) Circulating tumorcells (CTCs) are arrested in lung micro-vessels and escape from the blood byextravasation. CTC-derived materials are generated within minutes of CTC entry (29) and are captured by inflammatory monocytes inthe lung parenchyma. Inflammatory monocytes are activated and secretechemokines and cytokines. (C) Cytokines and chemokines secreted by inflammatorymonocytes promote the recruitment of both NK and T cells to the metastatic siteand either the killing of pioneer metastatic cells or the inhibition of theirdivision. In EE mice, reduced corticosterone levels result in an increased secretionof CCL20, CXCL2, CXCL10, IL-1b and TNF-a by inflammatory monocytes. The extravasationand activity of NK and T cells are enhanced resulting in increased tumor cellkilling. Red arrows indicate levels in EE mice relative to SE mice.Adrenocorticotropic hormone (ACTH); Corticotropic releasing hormone (CRH). **Table S1.** RNA-seq transcriptomic profiling of CMRA^+^ andCMRA^-^ Ly6C^+^ cells.SE and EE mice injected with CMRA-labelledB16:F10 cells. CMRA^+^ and CMRA^-^ Ly6C^+^ cellswere sorted 6 hours later and analyzed by RNA-seq transcriptomic profiling.List of genes differentially expressed between CMRA^+^ and CMRA^-^cells in SE and EE mice respectively. Log2-foldchanges and FDR are indicated. **Table S2.** Top canonical pathways of genes differentially expressedbetween CMRA^+^ and CMRA^-^ Ly6C^+^ cells.Ingenuity pathway analysis of genesdifferentially expressed in CMRA^+^ and CMRA^-^ Ly6C^+^cells. The names of the top canonical pathways are indicated as well as theproportions of genes defining the CMRA signature that belong to each pathway.The indicated p-values and z-scores refers to the statistical significance ofeach pathway

## Data Availability

The data sets generated during the current study are available from the corresponding author on reasonable request. Readers will be able to access the RNAseq data at XXXX.
