# Supplementary material for: Environmental signals perceived by the brain abate pro-metastatic monocytes by dampening glucocorticoids receptor signaling
Source: Cancer Cell Int. 2023 Feb 1;23:15. doi: 10.1186/s12935-023-02855-4 (PMC9893572; doi:10.1186/s12935-023-02855-4)
Supplement: Supplementary file 1 — Additional file 1: Fig. S1. Behavior and adult hippocampal neurogenesis in SE and EE mice.SE and EE mice were analyzed using the openfield test (A), the light and dark paradigm test (B), the Novelty SuppressedFeeding (NSF) test, (C) the Forced Swimming Test (FST) (D), and the Barnes mazetest (E). (A) Time spent in the aversive center of the open field arena inseconds (left). Number of entries in the central area (right). (B) Time spentin light. (C) Latency to eat. (D) Immobility time. (E) Spatial learning curveestablished by training in the Barnes maze for four consecutive days. (F) Numberof BrdU-positive nuclei in the dentate gyrus three weeks after BrdU treatment.Each dot represents a mouse (A-D, F). Mean ± s.e.m. of two experiments. *, p < 0.05; **, p < 0.01; ****, p < 0.0001. Fig. S2. Number of lymphoid and myeloid immune cell types in thesecondary lymphoid organs of SE and EE mice before tumor cell injection.C57BL/7 mice were housed under SE or EEconditions for 10-12 weeks and cell suspensions were prepared from the spleen(A, B) and mesenteric LN (C). (A) CD4+ T lymphocytes (CD4+CD3+), CD8+ T lymphocytes (CD8+ CD3+),B lymphocytes (CD19+ CD3-), NK T cells (NK1.1+CD3+), NK cells (NK1.1+ CD3-), neutrophils(CD11b+ CD11c- F4/80-Ly6C- Ly6Ghigh),CD11b+ DCs (CD11b+CD11c+), CD8+DCs (CD8+CD11c+), plasmacytoid DCs (CD11c+CD11b-120G8+),naïve CD4+ T cells (CD4+ CD3+ CD44low,CD62Lhigh), effector/memory CD4+ T cells (CD4+CD3+ CD44high) , regulatory CD4+ T cells (CD4+FoxP3+), naive CD8+ T cells (CD8+ CD3+CD44low, CD62Lhigh), memory CD8+ T cells (CD8+CD3+ CD44high) were identified by flow cytometry based onthe indicated surface markers. (A) Number of lymphoid cell types in spleennormalized to the mean values determined in SE mice. (B) Number of myeloid celltypes in spleen normalized to the mean values determined in SE mice. (C) Numberof lymphoid cell types in mesenteric LN normalized to the mean valuesdetermined in SE mice. Mean ±s.e.m. of two experiments. Fig. S3. Cytokine and [file 12935_2023_2855_MOESM1_ESM.docx]

**Additional file figures**

**Figure S1: Behavior and adult hippocampal neurogenesis in SE and EE mice**

SE and EE mice were analyzed using the open field test (A), the light and dark paradigm test (B), the Novelty Suppressed Feeding (NSF) test, (C) the Forced Swimming Test (FST) (D), and the Barnes maze test (E). (A) Time spent in the aversive center of the open field arena in seconds (left). Number of entries in the central area (right). (B) Time spent in light. (C) Latency to eat. (D) Immobility time. (E) Spatial learning curve established by training in the Barnes maze for four consecutive days. (F) Number of BrdU-positive nuclei in the dentate gyrus three weeks after BrdU treatment. Each dot represents a mouse (A-D, F). Mean ± s.e.m. of two experiments. *, p < 0.05; **, p < 0.01; ****, p < 0.0001.

**Figure S2: Number of lymphoid and myeloid immune cell types in the secondary lymphoid organs of SE and EE mice before tumor cell injection.**

C57BL/7 mice were housed under SE or EE conditions for 10-12 weeks and cell suspensions were prepared from the spleen (A, B) and mesenteric LN (C). (A) CD4^+^ T lymphocytes (CD4^+^ CD3^+^), CD8^+^ T lymphocytes (CD8^+^ CD3^+^), B lymphocytes (CD19^+^ CD3^-^), NK T cells (NK1.1^+^ CD3^+^), NK cells (NK1.1^+^ CD3^-^), neutrophils (CD11b^+^ CD11c^-^ F4/80^-^ Ly6C^-^ Ly6G^high^), CD11b^+^ DCs (CD11b^+^ CD11c^+^), CD8^+^ DCs (CD8^+^ CD11c^+^), plasmacytoid DCs (CD11c^+^CD11b^-^120G8^+^), naïve CD4^+^ T cells (CD4^+^ CD3^+^ CD44^low^, CD62L^high^), effector/memory CD4^+^ T cells (CD4^+^ CD3^+^ CD44^high^) , regulatory CD4^+^ T cells (CD4^+^ FoxP3^+^), naive CD8^+^ T cells (CD8^+^ CD3^+^ CD44^low^, CD62L^high^), memory CD8^+^ T cells (CD8^+^ CD3^+^ CD44^high^) were identified by flow cytometry based on the indicated surface markers. (A) Number of lymphoid cell types in spleen normalized to the mean values determined in SE mice. (B) Number of myeloid cell types in spleen normalized to the mean values determined in SE mice. (C) Number of lymphoid cell types in mesenteric LN normalized to the mean values determined in SE mice. Mean ± s.e.m. of two experiments.

**Figure S3: Cytokine and chemokine levels and frequency of immune cell types in the lungs of SE and EE mice before tumor cell injection.**

C57BL/7 mice were housed under SE or EE conditions for 10-12 weeks. (A, B) Lung protein extracts were prepared and the levels of the indicated cytokines (A) and chemokines (B) were measured. (C, D) Lung cell suspensions were prepared and the number of NK cells (NK1.1^+^ CD3^-^), T lymphocytes (CD19^-^ CD3^+^), B lymphocytes (CD19^+^ CD3^-^), inflammatory monocytes (Siglec-F^-^ CD11c^-^ Ly6G^-^ CD11b^high^ MHCII^-^ CD64^-^ Ly6C^+^), patrolling monocytes (Siglec-F^-^ CD11c^-^ Ly6G^-^ CD11b^high^ MHCII^-^ CD64^-^ Ly6C^-^), neutrophils (Siglec-F^-^ CD11c^-^ CD103^-^ CD11b^+^ Ly6G^high^), CD11b^+^ DCs (Siglec-F^-^ CD11c^+^ CD11b^high^ MHCII^+^ CD64^-^ CD24^+^), CD103^+^ DCs (Siglec-F^-^ CD11b^-^ CD103^+^ CD11c^+^ CD24^+^), interstitial macrophages (Siglec-F^-^ CD11c^-^ CD11b^high^ MHCII^+^ CD64^+^ CD24^-^), alveolar macrophages (CD11b^-^ Siglec-F^+^ CD11c^+^ CD64^+^) was determined by flow cytometry based on the indicated surface markers. (A) Cytokine levels in lung normalized to the mean values determined in SE mice. (B) Chemokine levels in lung normalized to the mean values determined in SE mice. (C) Number of myeloid cell types in lung normalized to the mean values determined in SE mice. (D) Number of lymphoid cell types in lung normalized to the mean values determined in SE mice. Mean ± s.e.m. of two experiments.

**Figure S4: Phenotypic characterization of LysM-Cre^+^:GR^fl/fl^and LysM-Cre^+^:** **Stop^fl/+^TdTomato**  **transgenic mice**

(A) Lung cells from LysM-Cre^+^:Stop^fl/+^TdTomato mice were analyzed by flow cytometry after gating on CD45^+^ cells and the frequencies of TdTomato^+^ cells among alveolar macrophages (CD11b^low^ CD11c^+^ CD64^+^ Ly6C^-^ Ly6G^-^ Siglec-F^+^), interstitial macrophages (CD11b^+^ CD11c^+^ CD24^-^ Ly6C^+^ Ly6G^-^ MHCII^+^ Siglec-F^-^), neutrophils (CD11b^+^ CD11c^-^ F4/80^-^ Ly6C^-^ Ly6G^high^), inflammatory monocytes (CD11b^+^ CD11c^-^ CD24^-^ Ly6C^high^ Ly6G^-^ MHCII^+/-^) patrolling monocytes (CD11b^+^ CD11c^med^ CD24^-^ Ly6C^low^ Ly6G^-^ MHCII^-^ Siglec-F^-^), DCs (CD11c^+^ Ly6C^-^ Ly6G^-^), and T lymphocytes (CD3^+^), were determined. Frequency of TdTomato^+^ cells among the indicated cell types in a representative mouse. (B) Peritoneal macrophages from LysM-Cre^+^:GR^fl/fl^  and GR^loxP/loxP^  mice were incubated with LPS in the presence of the indicated concentrations of corticosterone. Cellular supernatants were assessed for TNF-α secretion 24 hours later. Percentage of inhibition of TNF-α secretion relative to cells incubated in the absence of corticosterone. Mean ± s.e.m. of 4 mice/group. (C) Lung, spleen and blood cells from LysM-Cre^+^:Stop^fl/+^TdTomato and GR^loxP/loxP^  mice were analyzed by flow cytometry after gating on CD45^+^ cells for all immune cells and CD45^-^ cells for endothelial cells. Mean ± s.e.m. of 4 mice/group.

**Figure S5: Behavioral characterization of LysM-Cre^+^:GR^fl/fl^**

(A-D) GR^loxP/loxP^  and LysM-Cre^+^:GR^fl/fl^  mice were housed for 10 weeks under SE or EE conditions and analyzed using the open field test (A), the light and dark paradigm test (B), the Novelty Suppressed Feeding (NSF) test (C) and the forced swimming test (FST) (D). (A) Time spent in the aversive center of the open field arena. (B) Time spent in light. (C) Latency to eat. (D) Immobility time. Mean ± s.e.m.; **, p < 0.01; ***, p < 0.001; ****, p < 0.0001.

**Figure S6: Flow cytometry gating strategy used for analyzing lung-infiltrating immune cells and immunophenotyping of Ly6C-depleted mice.**

(A) Flow cytometry gating strategy. After isolation, lung cells were stained with 7-AAD and mAbs to CD11b, CD11c, CD24, CD45, CD64, CD103, F4/80, Ly6C, Ly6G, MHCII, Siglec-F and analyzed by flow cytometry. The gating strategy for identifying alveolar macrophages (AM)(CD11b^-^ Siglec-F^+^ CD11c^+^ CD64^+^), CD103^+^ DCs (Siglec-F^-^ CD11b^-^ CD103^+^ CD11c^+^ CD24^+^), neutrophils (Siglec-F^-^ CD11c^-^ CD103^-^ CD11b^+^ Ly6G^high^), inflammatory monocytes (iMo) (Siglec-F^-^ CD11c^-^ Ly6G^-^ CD11b^high^ MHCII^-^ CD64^-^ Ly6C^+^), patrolling monocytes (pMo) (Siglec-F^-^ CD11c^-^ Ly6G^-^ CD11b^high^ MHCII^-^ CD64^-^ Ly6C^-^), interstitial macrophages (IM) (Siglec-F^-^ CD11c^-^ CD11b^high^ MHCII^+^ CD64^+^ CD24^-^) and CD11b^+^ DCs (Siglec-F^-^ CD11c^+^ CD11b^high^ MHCII^+^ CD64^-^ CD24^+^) is shown. (B) Lung, slpeen and blood cells from Isotype-control  and anti-Ly6C-treated mice were analyzed by flow cytometry after gating either on CD45^+^ cells for all immune cells or CD45^-^ cells for endothelial cells. It is to be noted that anti-Ly6C clone Monts-1 was used as depleting antibody whereas anti-Ly6C clone AL-21 was used for flow cytometry staining. Mean ± s.e.m. of 6 mice/group. ***, p < 0.001; n.s. not significant.

**Figure S7: Flow cytometry analysis of Ly6C^+^ cells in lung.**

After isolation, lung cells from wt mice were stained with mAbs to CD103, CD11b, CD11c, CD24, CD45, Ly6C, Ly6G, MHCII and analyzed by flow cytometry after gating on CD45^+^ Ly6C^+^ alive cells. Representative FACS profiles and proportions of cells within the indicated gates.

**Figure S8: Cytokine serum levels 4 days after tumor cell injection**

C57BL/7 mice were housed under SE or EE conditions for 10-12 weeks. (A) Mice were injected with B16:F10 cells into the tail vein and serum samples were analyzed 4 days later for IFN-γ, IL-1β, IL-6, TNF-α, and IL-10 levels. (B) Mice were treated with either an anti-Ly6C or and isotype control mAb and injected one day after with B16:F10 cells into the tail vein. Serum samples were analyzed 4 days later for IFN-γ, IL-1β, IL-6, TNF-α, and IL-10 levels. Cytokine levels in individual mice normalized to the mean values determined in SE mice. Mean ± s.e.m. of three (A) or two (B) experiments. *, p < 0.05; **, p < 0.01; ***, p < 0.001; ****, p < 0.001.

**Figure S9: Differential transcriptomic, cytokine and chemokine profiles of inflammatory monocytes in EE and SE mice**

SE and EE mice were injected with CMRA-labelled B16:F10 cells and analyzed 6 hours later. (A-C) Lung cells were stained with DAPI and antibodies to CD45, CD11b and Ly6C, and analyzed by flow cytometry (A, C) or confocal microscopy after sorting of CMRA^+^ Ly6C^high^ cells (B). (A) Gating strategy. (B) Confocal analysis of representative CMRA^+^ Ly6C^high^ cells purified from SE mice after staining with anti-CD45 mAb (green). Arrows indicate CMRA^+^ cytoplasmic vesicles (red). (C) Number of CMRA^+^ inflammatory monocytes in individual SE and EE mice.

**Figure S10: Percentage of CMRA^+^ cells in SE and EE mice**

C57BL/7 mice were housed under SE or EE conditions for 10-12 weeks and injected with CMRA-labeled B16:F10 cells. Lung cell suspensions were prepared 16 hours later and the percentage of CMRA^+^ cells was measured after gating on neutrophils (Siglec-F^-^ CD11c^-^ CD103^-^ CD11b^+^ Ly6G^high^), inflammatory monocytes (Siglec-F^-^ CD11c^-^ Ly6G^-^ CD11b^high^ MHCII^-^ CD64^-^ Ly6C^+^), patrolling monocytes (Siglec-F^-^ CD11c^-^ Ly6G^-^ CD11b^high^ MHCII^-^ CD64^-^ Ly6C^-^), interstitial macrophages (Siglec-F^-^ CD11c^-^ CD11b^high^ MHCII^+^ CD64^+^ CD24^-^) and CD11b^+^ DCs (Siglec-F^-^ CD11c^+^ CD11b^high^ MHCII^+^ CD64^-^ CD24^+^). Percentage of CMRA^+^ in individual mice normalized to the mean values determined in SE mice. Mean ± s.e.m. two experiments.

**Figure S11: Differential transcriptomic profiles of inflammatory monocytes in EE and SE mice**

SE and EE mice were injected with CMRA-labelled B16:F10 cells and analyzed 6 hours later. Lung cells were stained with DAPI and antibodies to CD45, CD11b and Ly6C. CMRA^+^ and CMRA^-^ Ly6C^high^ cells were sorted and analyzed by RNA sequencing. (A) Volcano plot analysis of genes differentially expressed in CMRA^+^ and CMRA^-^ Ly6C^+^ cells in SE (left) and EE (right) mice. (B) Scatter plot representation showing, for each transcript, the ratio between expression level in CMRA^+^ and CMRA^-^ cells in EE mice (Y axis) and in SE mice (X axis). (C) Clustering heat map of 16 samples based on the 289 genes differentially expressed between CMRA^+^ and CMRA^-^ cells in SE and EE mice 6 hours after tumor cell injection.

**Figure S12: Transcriptomic analysis of Ly6C^+^ cells from LysM-Cre^+^:GR^fl/fl^** **mice**

LysM-Cre^+^:GR^fl/fl^ mice and GR^loxP/loxP^ were housed for 10-12 weeks under SE or EE conditions, and injected with CMRA-loaded B16:F10 cells into the tail vein. Lung cell suspensions were prepared 4 days later, and Ly6C^+^ cells were sorted by flow cytometry and analyzed by RNA-seq transcriptomic profiling. Expression levels of CCL3, CCL4, Fos, Rel and IL-1β in individual mice. Mean ± s.e.m. of 8 mice. *, p < 0.05; **, p < 0.01; ***, p < 0.001.

**Figure S13: Schematic representation of the impact of the housing environment on the immune system during the late stages of lung metastasis**

(A) Schematic representation of the impact of enriched environment on the hypothalamic-pituitary-adrenal axis. Sensory, cognitive and motor stimuli are integrated in the hypothalamus. Glucocorticoid production by adrenal glands is regulated by the adrenocorticotropic hormone (ACTH), which is secreted by the anterior pituitary gland in response to corticotropin-releasing hormone (CRH) produced in the hypothalamus. In EE mice, the activity (and weight) of adrenal glands is decreased resulting in lower corticosterone serum levels. (B, C) Schematic representation of the interactions between immune and tumor cells in the lung microenvironment 0-6 hours (B) and 3-4 days (C) after B16:F10 cell injection. (B) Circulating tumor cells (CTCs) are arrested in lung micro-vessels and escape from the blood by extravasation. CTC-derived materials are generated within minutes of CTC entry (29) and are captured by inflammatory monocytes in the lung parenchyma. Inflammatory monocytes are activated and secrete chemokines and cytokines. (C) Cytokines and chemokines secreted by inflammatory monocytes promote the recruitment of both NK and T cells to the metastatic site and either the killing of pioneer metastatic cells or the inhibition of their division. In EE mice, reduced corticosterone levels result in an increased secretion of CCL20, CXCL2, CXCL10, IL-1β and TNF-α by inflammatory monocytes. The extravasation and activity of NK and T cells are enhanced resulting in increased tumor cell killing. Red arrows indicate levels in EE mice relative to SE mice. Adrenocorticotropic hormone (ACTH); Corticotropic releasing hormone (CRH).

**Table S1: RNA-seq transcriptomic profiling of CMRA^+^ and CMRA^-^ Ly6C^+^ cells**

SE and EE mice injected with CMRA-labelled B16:F10 cells. CMRA^+^ and CMRA^-^ Ly6C^+^ cells were sorted 6 hours later and analyzed by RNA-seq transcriptomic profiling. List of genes differentially expressed between CMRA^+^ and CMRA^-^ cells in SE and EE mice respectively. Log2-fold changes and FDR are indicated.


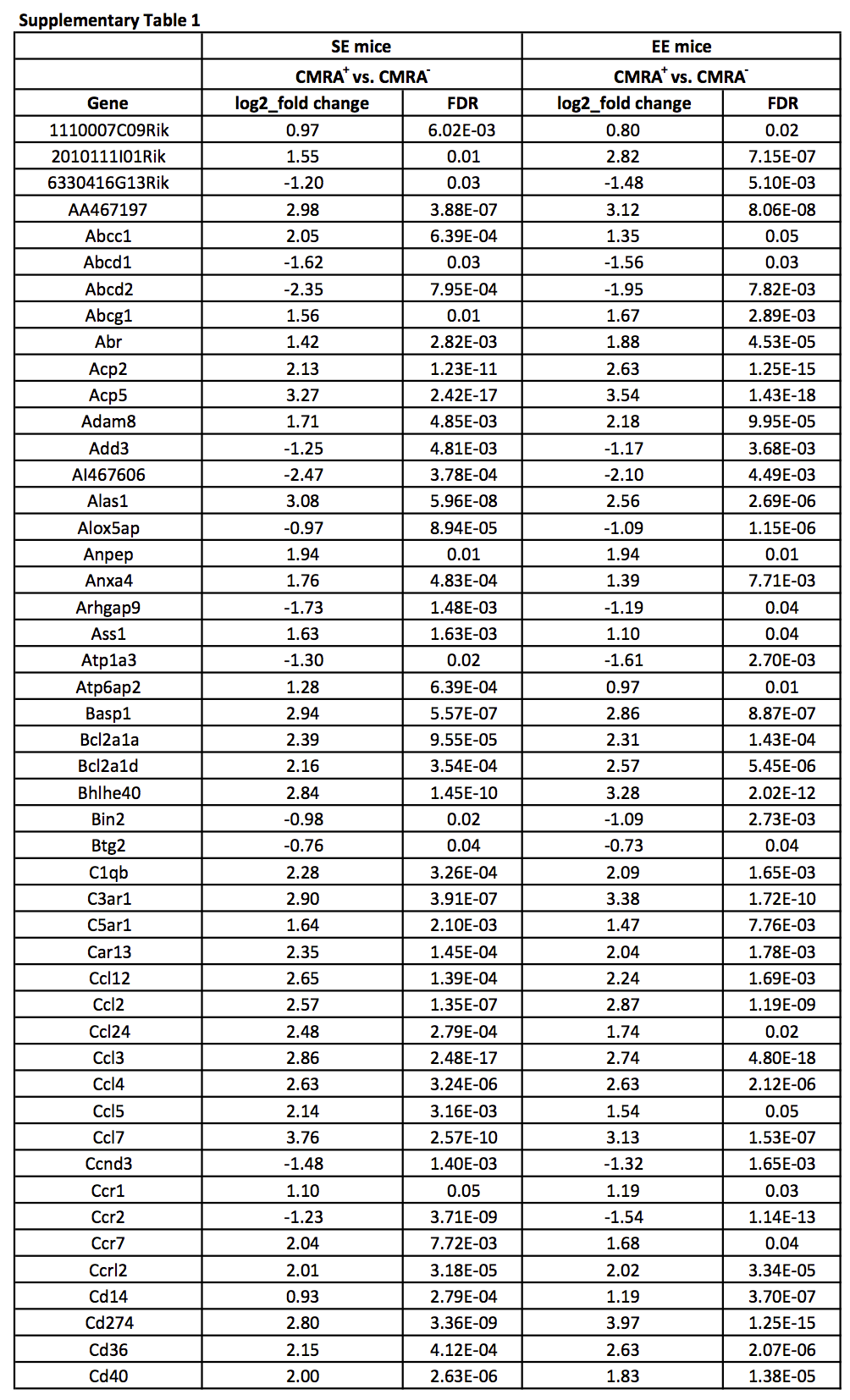


**
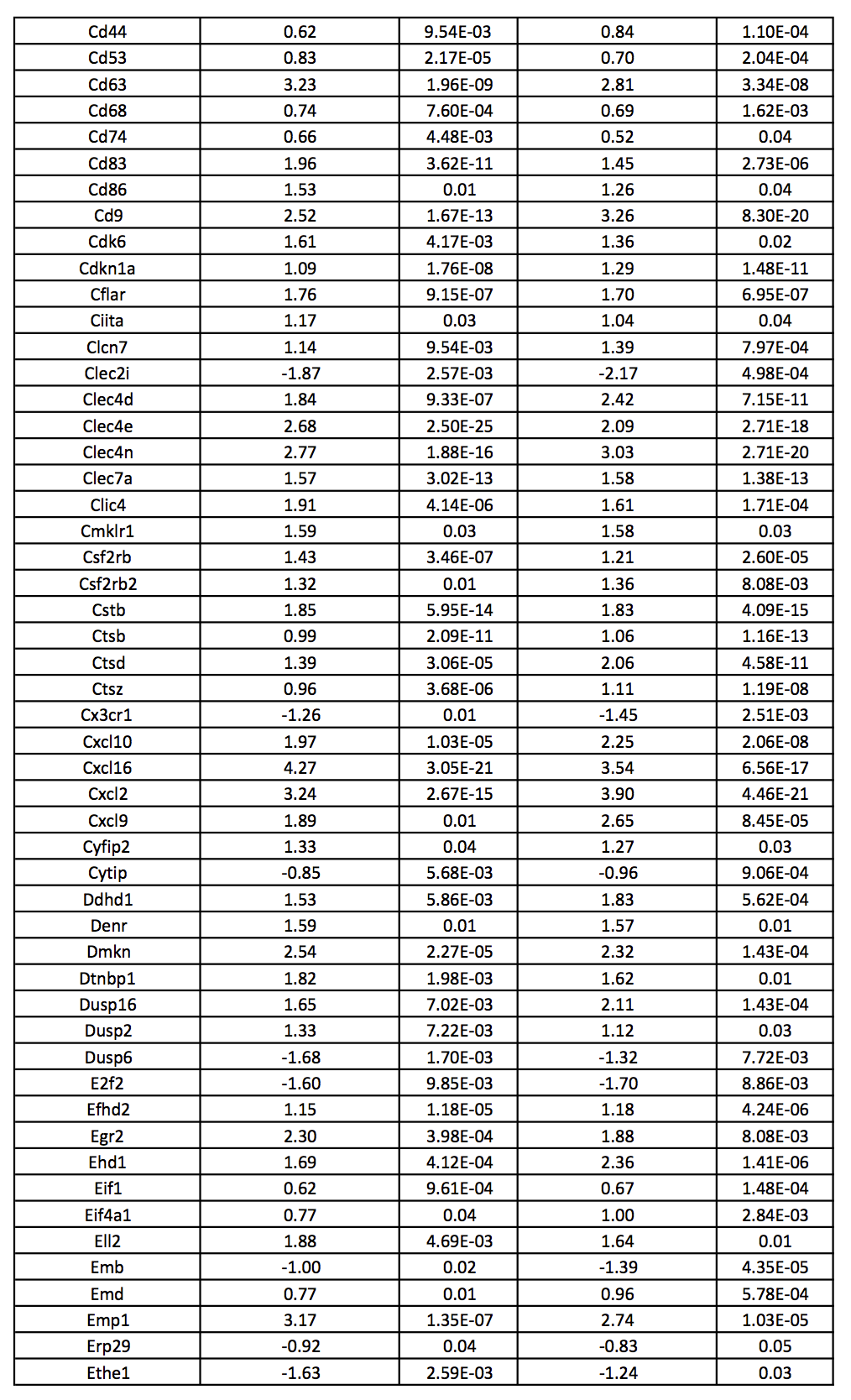
** **
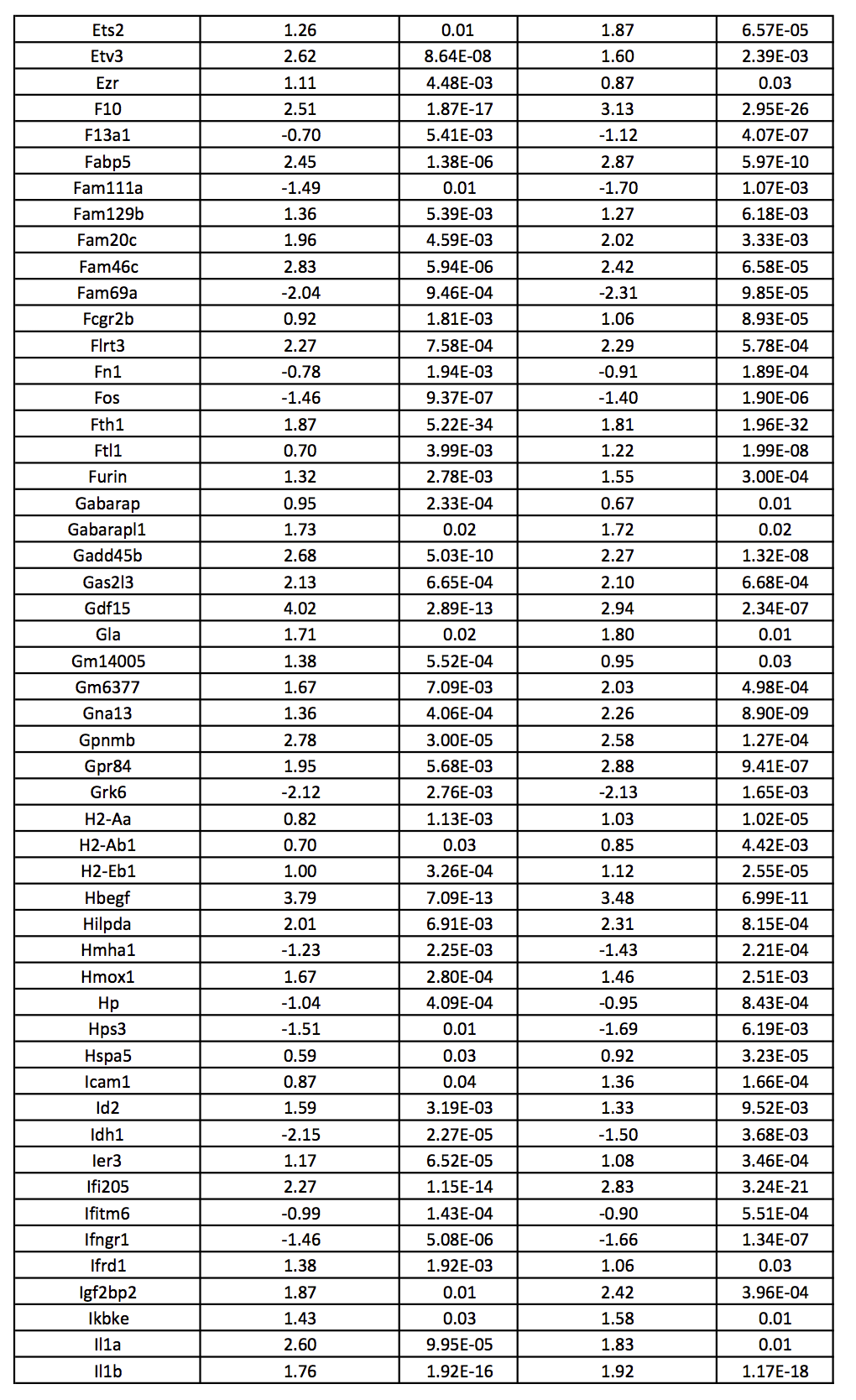
**
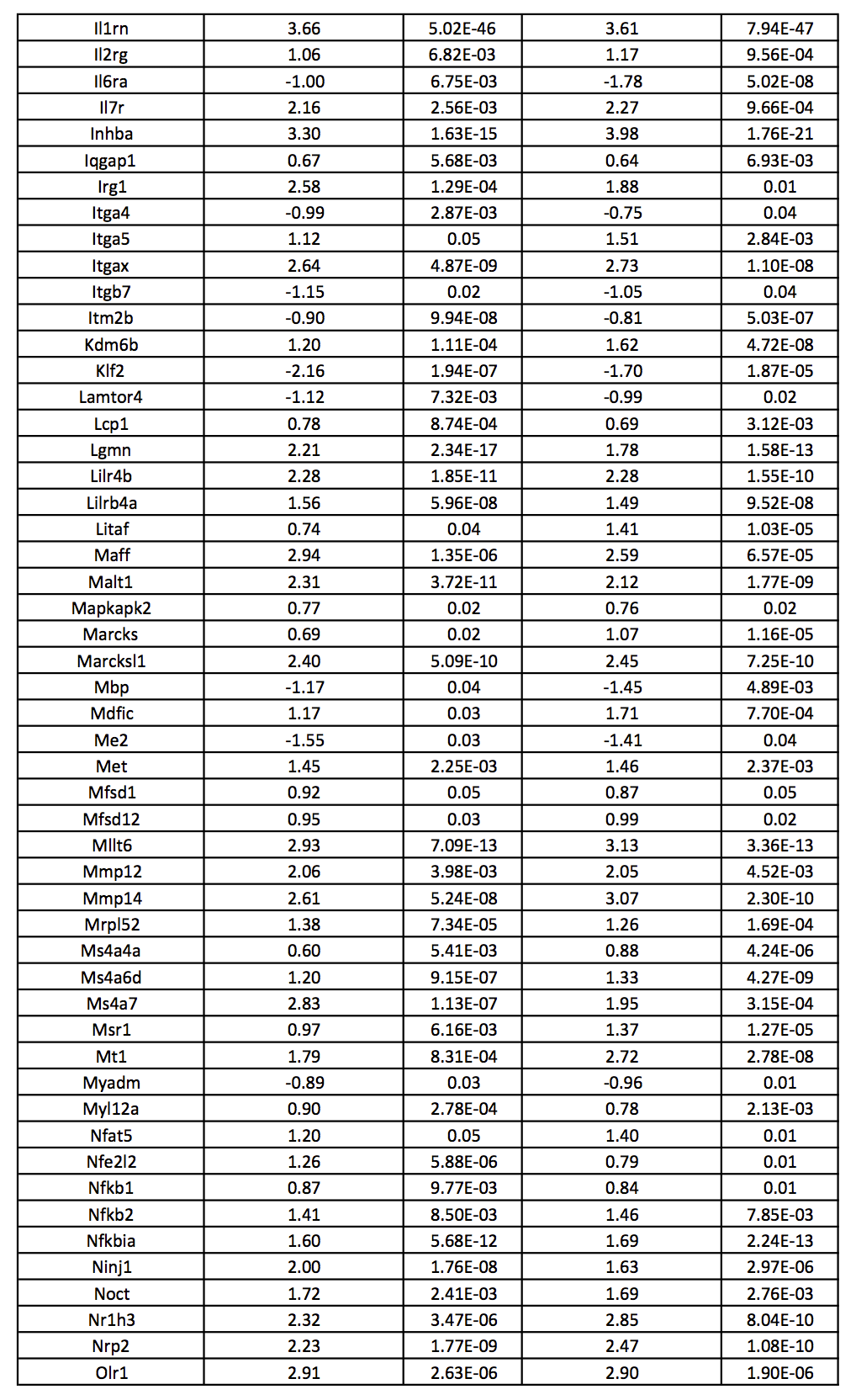

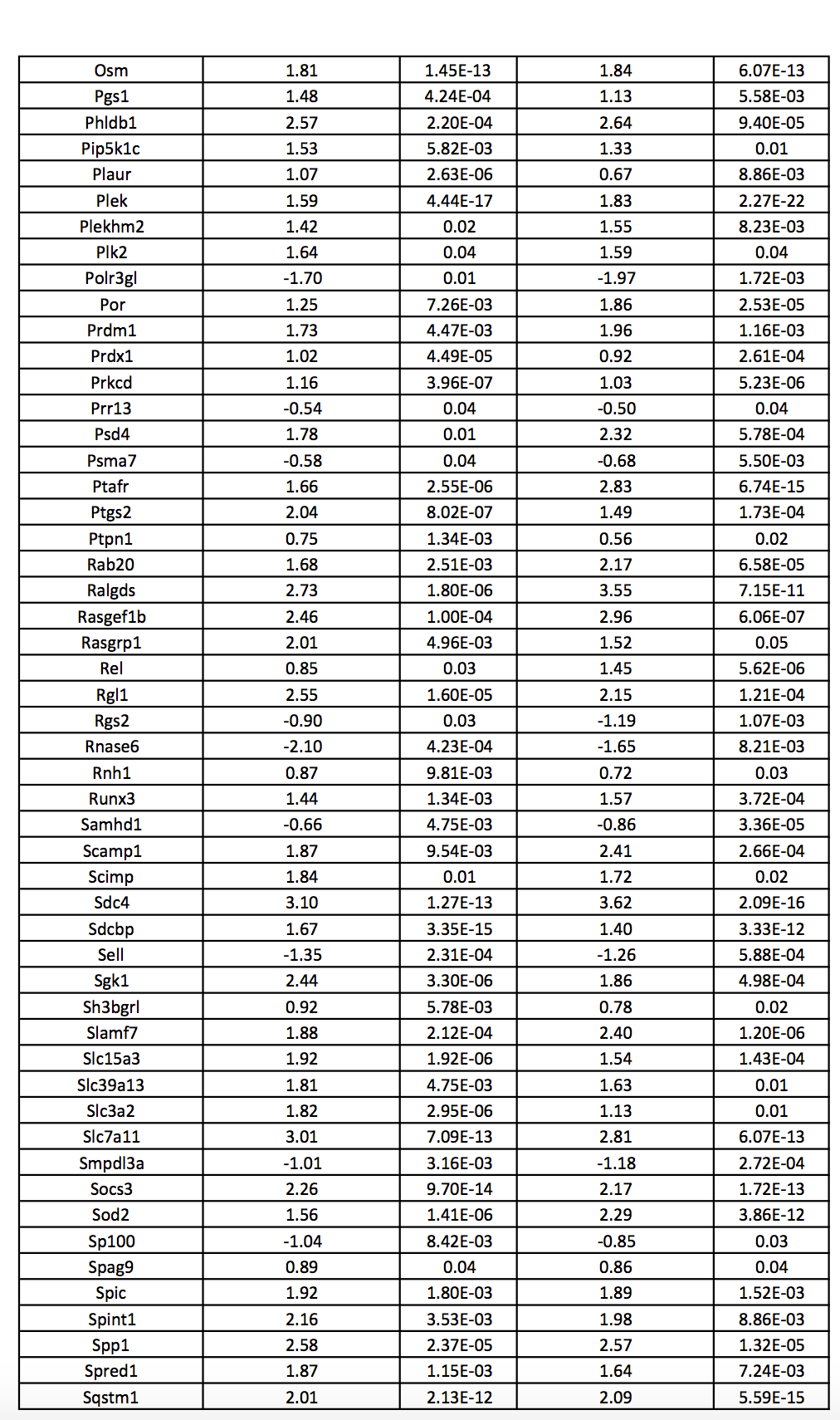

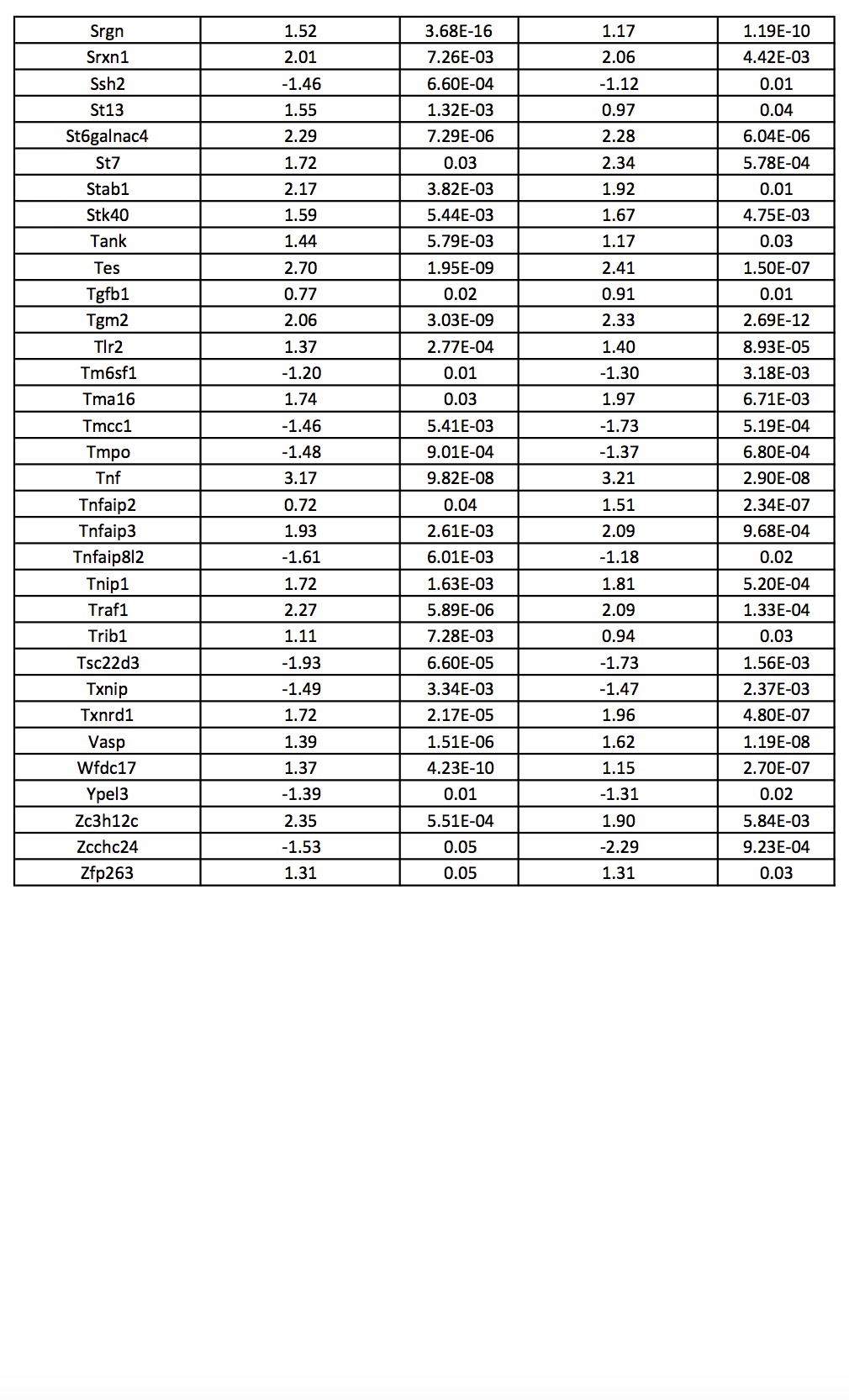


**Table S2: Top canonical pathways of genes differentially expressed between CMRA^+^ and CMRA^-^ Ly6C^+^ cells**

Ingenuity pathway analysis of genes differentially expressed in CMRA^+^ and CMRA^-^ Ly6C^+^ cells. The names of the top canonical pathways are indicated as well as the proportions of genes defining the CMRA signature that belong to each pathway. The indicated p-values and z-scores refers to the statistical significance of each pathway.

**
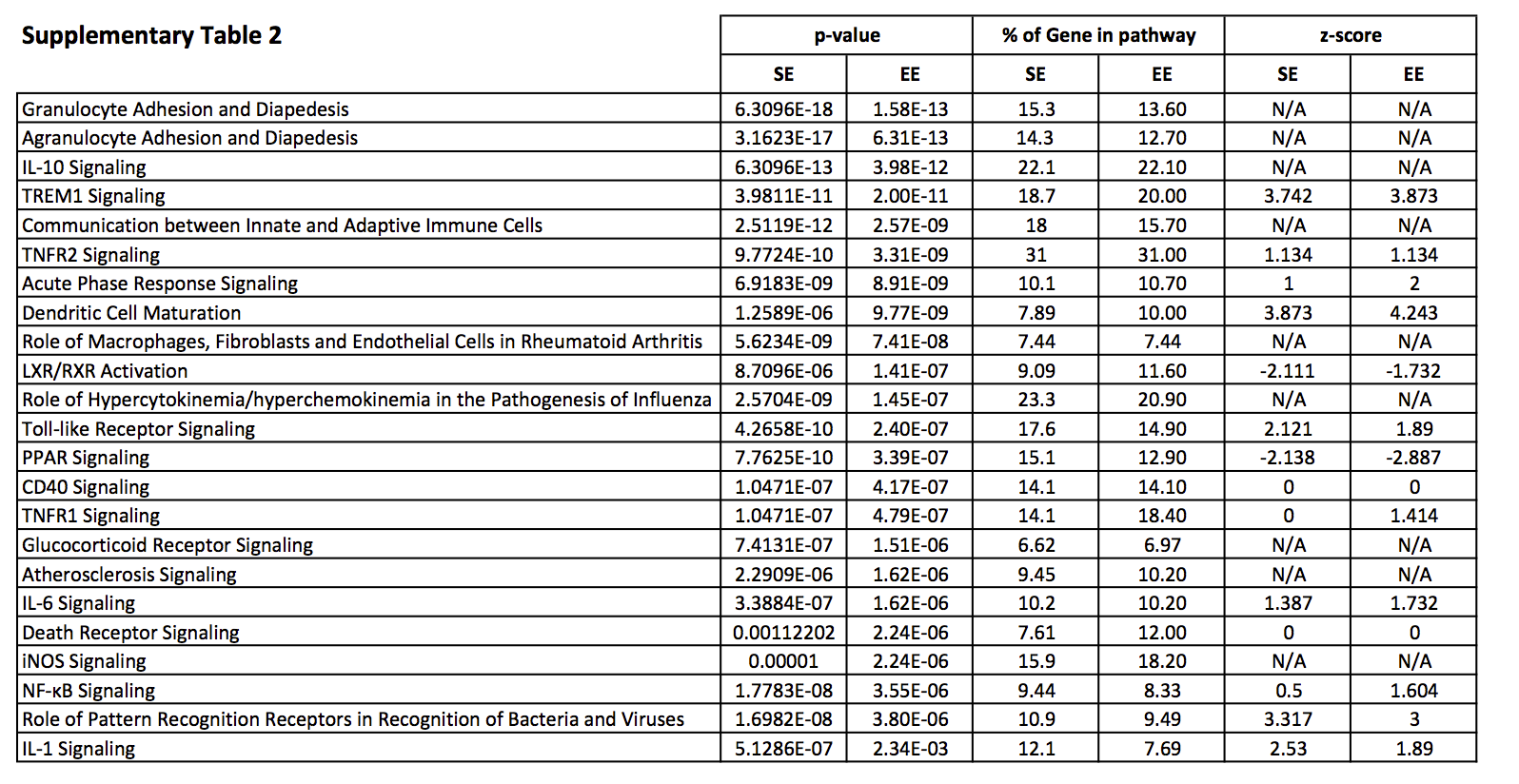
**
